# Osteomodulin attenuates smooth muscle cell osteogenic transition in vascular calcification

**DOI:** 10.1002/ctm2.682

**Published:** 2022-02-20

**Authors:** Nikolaos T. Skenteris, Till Seime, Anna Witasp, Eva Karlöf, Grzegorz B. Wasilewski, Marina A. Heuschkel, Armand M.G. Jaminon, Loureen Oduor, Robert Dzhanaev, Malin Kronqvist, Mariette Lengquist, Frederique E.C.M. Peeters, Magnus Söderberg, Rebecka Hultgren, Joy Roy, Lars Maegdefessel, Hildur Arnardottir, Eva Bengtsson, Isabel Goncalves, Thomas Quertermous, Claudia Goettsch, Peter Stenvinkel, Leon J. Schurgers, Ljubica Matic

**Affiliations:** ^1^ Cardiovascular Medicine Unit Department of Medicine Karolinska Institute Stockholm Sweden; ^2^ Division of Vascular Surgery Department of Molecular Medicine and Surgery Karolinska Institute Stockholm Sweden; ^3^ Department of Biochemistry and CARIM School for Cardiovascular Diseases Maastricht University Maastricht Netherlands; ^4^ Division of Renal Medicine Department of Clinical Sciences Intervention and Technology Karolinska Institute Stockholm Sweden; ^5^ Nattopharma ASA, Oslo Norway; ^6^ Department of Internal Medicine I‐Cardiology Medical Faculty RWTH Aachen University, Aachen, Germany; ^7^ Department of Clinical Sciences Malmö and Cardiology Skåne University Hospital Lund University Lund Sweden; ^8^ Biointerface Group Helmholtz Institute for Biomedical Engineering RWTH Aachen University Aachen Germany; ^9^ Department of Cardiology and CARIM School for Cardiovascular Diseases Maastricht University Medical Center Maastricht Netherlands; ^10^ Cardiovascular Renal and Metabolism Safety Clinical Pharmacology and Safety Sciences R&D, AstraZeneca Gothenburg Sweden; ^11^ Klinikum rechts der Isar Department for Vascular and Endovascular Surgery Technical University Munich Munich Germany; ^12^ Department of Cardiovascular Medicine, University of Stanford Stanford California USA; ^13^ Institute of Experimental Medicine and Systems Biology RWTH Aachen University Aachen Germany

**Keywords:** aortic valves, atherosclerosis, calcification, chronic kidney disease, osteogenic transdifferentiation, osteomodulin, smooth muscle cells

## Abstract

**Rationale:**

Vascular calcification is a prominent feature of late‐stage diabetes, renal and cardiovascular disease (CVD), and has been linked to adverse events. Recent studies in patients reported that plasma levels of osteomodulin (OMD), a proteoglycan involved in bone mineralisation, associate with diabetes and CVD. We hypothesised that OMD could be implicated in these diseases via vascular calcification as a common underlying factor and aimed to investigate its role in this context.

**Methods and results:**

In patients with chronic kidney disease, plasma OMD levels correlated with markers of inflammation and bone turnover, with the protein present in calcified arterial media. Plasma OMD also associated with cardiac calcification and the protein was detected in calcified valve leaflets by immunohistochemistry. In patients with carotid atherosclerosis, circulating OMD was increased in association with plaque calcification as assessed by computed tomography. Transcriptomic and proteomic data showed that OMD was upregulated in atherosclerotic compared to control arteries, particularly in calcified plaques, where OMD expression correlated positively with markers of smooth muscle cells (SMCs), osteoblasts and glycoproteins. Immunostaining confirmed that OMD was abundantly present in calcified plaques, localised to extracellular matrix and regions rich in α‐SMA^+^ cells. In vivo, OMD was enriched in SMCs around calcified nodules in aortic media of nephrectomised rats and in plaques from *ApoE* ^−/−^ mice on warfarin. In vitro experiments revealed that OMD mRNA was upregulated in SMCs stimulated with IFNγ, BMP2, TGFβ1, phosphate and β‐glycerophosphate, and by administration of recombinant human OMD protein (rhOMD). Mechanistically, addition of rhOMD repressed the calcification process of SMCs treated with phosphate by maintaining their contractile phenotype along with enriched matrix organisation, thereby attenuating SMC osteoblastic transformation. Mechanistically, the role of OMD is exerted likely through its link with SMAD3 and TGFB1 signalling, and interplay with BMP2 in vascular tissues.

**Conclusion:**

We report a consistent association of both circulating and tissue OMD levels with cardiovascular calcification, highlighting the potential of OMD as a clinical biomarker. OMD was localised in medial and intimal α‐SMA^+^ regions of calcified cardiovascular tissues, induced by pro‐inflammatory and pro‐osteogenic stimuli, while the presence of OMD in extracellular environment attenuated SMC calcification.

## Graphical abstract

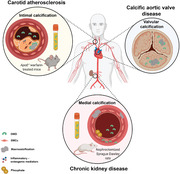

## 1 INTRODUCTION

Calcification is one of the key features of late‐stage human cardiovascular disease (CVD),^1^ associated with atherosclerosis,^2^ diabetes mellitus,^3^ chronic kidney disease (CKD),^4^ and calcific aortic valve disease (CAVD).^5^ Calcification is an active pathophysiological process characterised by the deposition of calcium phosphate crystals in the form of hydroxyapatite, which occurs in the media or intima of arteries and aortic valve leaflets, contributing to vascular and valvular calcification, respectively. Vascular calcification linked with inflammation is the hallmark of disease progression and a strong independent prognostic risk marker for adverse coronary events.^6^ Coronary micro‐calcification has been linked to unstable atherosclerotic lesions in patients^7^ and plaque inflammation in *ApoE* ^−/−^ mice,^8^ by increasing stress levels within the fibrous cap leading to plaque rupture, thrombosis and myocardial infarction.^9^ In contrast, late‐stage carotid intimal macro‐calcification has been associated with a more stable molecular plaque phenotype, characterised by repressed inflammation and an increase in markers of typical smooth muscle cells (SMCs; i.e. SMA, MYH11, CNN1).^10^, ^11^ However, it has not yet been conclusively shown what is its long‐term impact on patient or lesion vulnerability. More detailed knowledge of the underlying molecular mechanisms associated with different types of calcification across vascular beds is needed and would be essential for better clinical management of CVD.

Currently, computed tomography (CT) angiography remains the sole non‐invasive detection method of calcification, where recent software developments for quantitative CT‐image‐based analyses have led to a better resolution in structural and morphological assessment of vascular lesions.^10^, ^12^ In recent years, unmet needs for a more accurate detection of vascular calcification have driven an intensive research on circulating biomarkers and their assessment for effective diagnosis of early calcification, as well as improved preventive and prognostic strategies.^13^ Such circulating biomarkers (i.e. FGF23, fetuin A, sclerostin, osteoprotegerin, matrix Gla protein, osteopontin, etc.)^14^ are important since they may also be advantageous as indicators of the therapeutically alleviated cardiovascular burden.^15^ In this regard, results from an unbiased plasma proteomic profiling of a large CVD cohort indicated osteomodulin (OMD, also termed osteoadherin) as one of the potential novel circulating biomarkers associated with cardiovascular risk traits^16^ and type 2 diabetes.^17^ OMD is a small leucine‐rich keratan sulphate proteoglycan found in the extracellular matrix (ECM)^18^ of mineralised tissues, such as bones^19^ and teeth,^20^ where it is typically expressed by RUNX2^+^ osteoblasts^21^, ^22^ and displays a high binding affinity to hydroxyapatite. However, the association of circulating OMD levels with CVD traits is novel^16^, ^17^ and has yet to be evaluated in independent cohorts, as well as its role in vascular tissue pathophysiology.

Considering that OMD has been linked with tissue mineralisation and that calcification is associated with diabetes and renal dysfunction in addition to CVD, we hypothesised that OMD could be a circulating biomarker for cardiovascular calcification in general. In the current study, we aimed to (i) evaluate OMD as a broad plasma biomarker of cardiovascular calcification and in relation to patient risk traits and (ii) investigate its expression and function in calcified vascular beds. We designed an integrative approach combining plasma and tissue profiling analysis from large human biobanks to assess OMD in (1) CKD5 patients selected for living donor kidney transplantation, (2) carotid stenosis patients undergoing stroke‐preventive carotid endarterectomy (CEA) and (3) aortic valve stenosis patients undergoing valve replacement (Visual Abstract). Functionally, we also studied OMD in two murine models of intimal and medial macro‐calcification, respectively, an *ApoE* ^−/−^ atherosclerotic mouse model on warfarin and a nephrectomised rat model. Finally, mechanistic in vitro studies explored the role of OMD in the calcification process. Our findings reveal both systemic and local tissue association between increased OMD levels and calcification in patients with atherosclerosis, CKD and CAVD, with an ultimately protective role of OMD in the ECM mineralisation via SMCs.

## 2 MATERIALS AND METHODS

Material and data pertaining to this manuscript are available from the corresponding author pending reasonable request. Restrictions associated with human biobank protection and personal data GDPR legislation will be respected.

1 **HIGHLIGHT**

- Vascular calcification is a feature of late‐stage diabetes, renal and cardiovascular disease, linked to major adverse events.
- Utilising patient cohorts, murine models and in vitro studies, we identified osteomodulin (OMD), proteoglycan involved in bone mineralisation, as a novel factor enriched in plasma and tissue in association with cardiovascular calcification.
- OMD is induced by inflammation and osteoblastic transition of smooth muscle cells (SMCs) and capable of attenuating matrix calcification, regulated by SMAD3 and TGFB1 signalling, and via interplay with BMP2 in vascular tissues.
- The potential of OMD as a clinical biomarker or therapeutic target for calcification should be investigated.

### 2.1 Human material

#### 2.1.1 Chronic kidney disease cohort

The CKD cohort comprised inferior epigastric arterial biopsies from CKD5 patients undergoing kidney transplant surgery at the Karolinska Hospital, Sweden. A subset of 98 patients were used from a prospective study ongoing since March 2009.^23^ All human samples of CKD cohort were collected with informed consent from patients or organ donors’ guardians; studies were approved by the regional Ethical Committee in Stockholm, part of the Swedish Ethical Review Authority, under the EPM approval (2008/1748‐31/2) and follow the guidelines of the Declaration of Helsinki. Patient selection was based on histological vascular media calcification score (as described below): no media calcification *n* = 25; minor media calcification *n* = 25; moderate media calcification *n* = 24; and severe calcification *n* = 24. The median age was 51 years (range 22–71 years) and 72% of the patients were males. The CKD study cohort demographics (Table S1) and details of sample processing were previously described.^24^ Patients were diagnosed with chronic glomerulonephritis (33%), polycystic kidney disease (10%), diabetic nephropathy (6%) and other or unknown etiologies (51%). The two predominant comorbid conditions were CVD (cerebrovascular, cardiovascular and/or peripheral vascular disease; 25%) and diabetes mellitus (19%). A majority of the patients were treated with phosphate binders (90%) and erythropoiesis‐stimulating agents (82%) and 42% were on statins. Antihypertensive medications included angiotensin‐converting enzyme inhibitors and/or angiotensin II receptor antagonists (55%), betablockers (58%) and calcium‐channel blockers (55%). In addition, 65% of the patients received peritoneal dialysis or hemodialysis before undergoing kidney transplantation.

Within 20 min after skin incision at the start of surgery, one piece (1–2 cm in length) of the inferior epigastric artery was collected by sharp dissection. Samples were divided and prepared for RNA isolation (preserved in All Protect Tissue Reagent, Qiagen, Hilden, Germany and stored at −80°C) or for immunohistochemistry (fixed in 4% phosphate‐buffered formalin and embedded in paraffin). The patient samples were assessed histologically with von Kossa stain, evaluated by experienced pathologists and scored according to grades of medial calcification, where score 0 represented no calcification, 1 corresponded to minimal calcification, 2 moderate and score 3 represented severe calcification.

#### 2.1.2 Clinical and biochemical variables in the CKD cohort

Fasting blood samples were collected prior to the surgical procedure and analysed for high‐sensitivity C‐reactive protein (hsCRP), triglycerides, cholesterol, high‐density lipoprotein (HDL), glucose, glycated haemoglobin (haemoglobin A1c or HBA1c), haemoglobin b, calcium (Ca), phosphate (PO_4_), magnesium (Mg), vitamin D metabolites (25(OH) D‐vitamin and 1,25(OH) D‐vitamin), with routine biochemical tests at the accredited Clinical Chemical Laboratory Lab of Karolinska University Hospital, Stockholm, Sweden. Samples that were not immediately used were stored at ‐70°C pending analyses. Interleukin 6 (IL‐6), tumour necrosis factor (TNF), thyroid‐stimulating hormone (TSH), free triiodothyronine (fT3), free thyroxine (fT4) and parathyroid hormone (PTH) were analysed by an immunometric assay on an Immulite 1000 Analyzer (Siemens Healthcare Diagnostics, Los Angeles, CA, USA). 8‐Hydroxy‐2′‐deoxyguanosine (8‐OHdG), alkaline phosphatase (ALP), klotho and sclerostin were analysed with commercial kits as previously described.^23^ Briefly, 8‐OHdG was measured using a commercial competitive enzyme‐linked immunosorbent assay (ELISA) kit (Japan Institute for the Control of Aging, Shizuoka, Japan). Total ALP activity was assessed using a commercial reagent kit (Alkaline Phosphatase (IFCC) Plus®, Thermo Fisher Scientific Oy, Vantaa, Finland), and analysed by an automatic chemical analyser (Konelab 20XTi®, Thermo Electron Corporation, Vantaa, Finland). Klotho was measured by Human soluble α‐Klotho ELISA Assay from IBL International (Hamburg, Germany). Human sclerostin was analysed with ELISA kits from R&D systems (Abingdon, UK). Plasma pentosidine was analysed by reverse‐phase high‐performance liquid chromatography (HPLC). The total (free plus protein bound) plasma pentosidine concentration measured in nmol/L was corrected for albumin and expressed as nmol of plasma pentosidine per gram of albumin. Advanced glycation end product (AGE) autofluorescence was measured using an autofluorescence reader (DiagnOptics BV, Groningen, Netherlands). Aortic valve calcification scores were computed using the Agatston coronary artery calcium (CAC) scoring method from non‐contrast cardiac CT scans, obtained and evaluated by radiologists according to protocols described elsewhere.^23^ Valve calcification was determined as the sum of total calcifications in the aortic valve area including calcifications within the valve leaflets as well in the aortic wall immediately connected to the leaflets.

#### 2.1.3 Carotid atherosclerosis cohort (Biobank of Karolinska Endarterectomies)

Patients undergoing surgery for high‐grade (>50% North American Symptomatic Carotid Endarterectomy Trial [NASCET])^25^ carotid stenosis at the Department of Vascular Surgery, Karolinska University Hospital were consecutively enrolled in the Biobank of Karolinska Endarterectomies (BiKE, Karolinska University Hospital) and clinical data recorded on admission. Symptoms (S) were defined as transitory ischemic attack (TIA), minor stroke (MS) and *amaurosis fugax* (retinal TIA). Patients without qualifying symptoms within 6 months prior to surgery were categorised as asymptomatic (AS) and indication for CEA based on results from the Asymptomatic Carotid Surgery Trial (ACST).^26^ All human samples were collected with informed consent from patients or organ donors’ guardians; studies were approved by the regional Ethical Committee with the following ethical permit numbers: BiKE EPN DNr 95‐276/277; 01‐199; 02‐146; 02‐147; 2009/295‐31/2; 2009/512‐31/2; 2011/950‐32; 2013/2137‐32; 2017/508‐32; 2018/954‐32. BiKE studies follow the guidelines of the Declaration of Helsinki.

Peripheral blood (10 ml) from patients (*n* = 85) was retrieved from peripheral arterial line at surgery. Samples were collected into ethylenediaminetetraacetic acid (EDTA)‐coated Vacutainer tubes (Becton‐Dickenson, San Jose, CA, USA) and centrifuged at 2400 *g* for 10 min at 4°C directly after sampling. Supernatants were transferred to cryotubes and stored at −80°C until analysed. Patients’ clinical characteristics are described in Table S2. Control subjects (*n* = 33) were all male, age 65 years, and had no medical history of cancer, acute myocardial infarction, angina pectoris, peripheral arterial occlusive disease, hypertension, or ongoing therapy with platelet aggregation inhibitors or statins. Carotid endarterectomies (carotid atherosclerotic plaques) were also collected at surgery. Briefly, plaques were divided transversally at the most stenotic part; the proximal half of the lesion was used for RNA preparation while the distal half was immediately fixed in 4% formaldehyde for 48 h. Non‐atherosclerotic, further referred to as normal artery, control samples (*n* = 10 in total) were obtained from macroscopically disease‐free iliac arteries. For the ex vivo culture assay, fresh carotid atherosclerotic plaques (*n* = 6) were collected in RPMI 1640 complete medium and processed upon arrival in the laboratory. The BiKE cohort demographics, details of sample collection and processing and transcriptomic analyses by microarrays were previously described in details^27^, ^28^, ^29^ and here briefly presented in Table S3.

#### 2.1.4 Calcific aortic valve disease cohort

In this prospective, cross‐sectional observational study, human aortic valves were obtained from 50 patients with aortic valve stenosis scheduled for (isolated or combined) aortic valve replacement at Maastricht University Medical Center+ (MUMC+), Maastricht, Netherlands.^30^ Clinical information was obtained from the electronic hospital charts and the study was approved by the local Institutional Review Board under the number METC‐16‐4‐031 and follow the guidelines of the Declaration of Helsinki. All patients provided informed consent prior to inclusion. Valvular tissue samples were transported from the operating room in paraformaldehyde 4% (PFA). Tissue samples were processed and embedded in paraffin (*n* = 40). Valve leaflets were sectioned in 5 μm sections using a Microtome (Leica Reichert Jung 2035, Germany) and collected on glass slides.

### 2.2 Molecular biology methods

#### 2.2.1 Secreted OMD protein measurements

Circulating OMD protein levels were quantified from patient EDTA‐plasma samples and the supernatants of ex vivo cultured plaques with the human OMD ELISA Kit (#OKEH03353, Aviva Systems Biology, San Diego, CA, USA) according to the manufacturer's protocol.

### 2.3 Statistical analyses

Distribution of the data was assessed using the Shapiro–Wilks normality test. Comparative statistics between normally distributed groups was performed using Student's *t*‐test, or one‐way ANOVA with Bonferroni multiple comparison post‐test. The Mann–Whitney or Kruskal–Wallis test with Dunn's multiple comparison test was used for comparison of two or multiple groups, respectively, when normal distribution assumption was invalid. Spearman and Pearson correlation coefficients were used to assess all correlations according to normality test result and Spearman correlation coefficient was used in univariate analysis of clinical variables in the CKD cohort. Multivariable linear regression analysis was used to estimate the significance between plasma OMD levels and plaque morphological features from CT assessment (Table S4). Pearson's chi‐squared test was used for comparing calcification score versus diabetes, smoking and statins in Table S1. Fisher's exact test was used to test significance of the categorical variables from demographic/clinical tables. Differences between groups were considered significant at *p*‐values < .05 (*p < .05, **p ≤ .01, ***p ≤ .001, ****p ≤ .0001). Data were analysed using Prism version 9.0 (GraphPad Software, Inc., San Diego, CA, USA).

## 3 RESULTS

### 3.1 OMD levels are increased with cardiovascular calcification in CKD and CAVD patients

**FIGURE 1:**
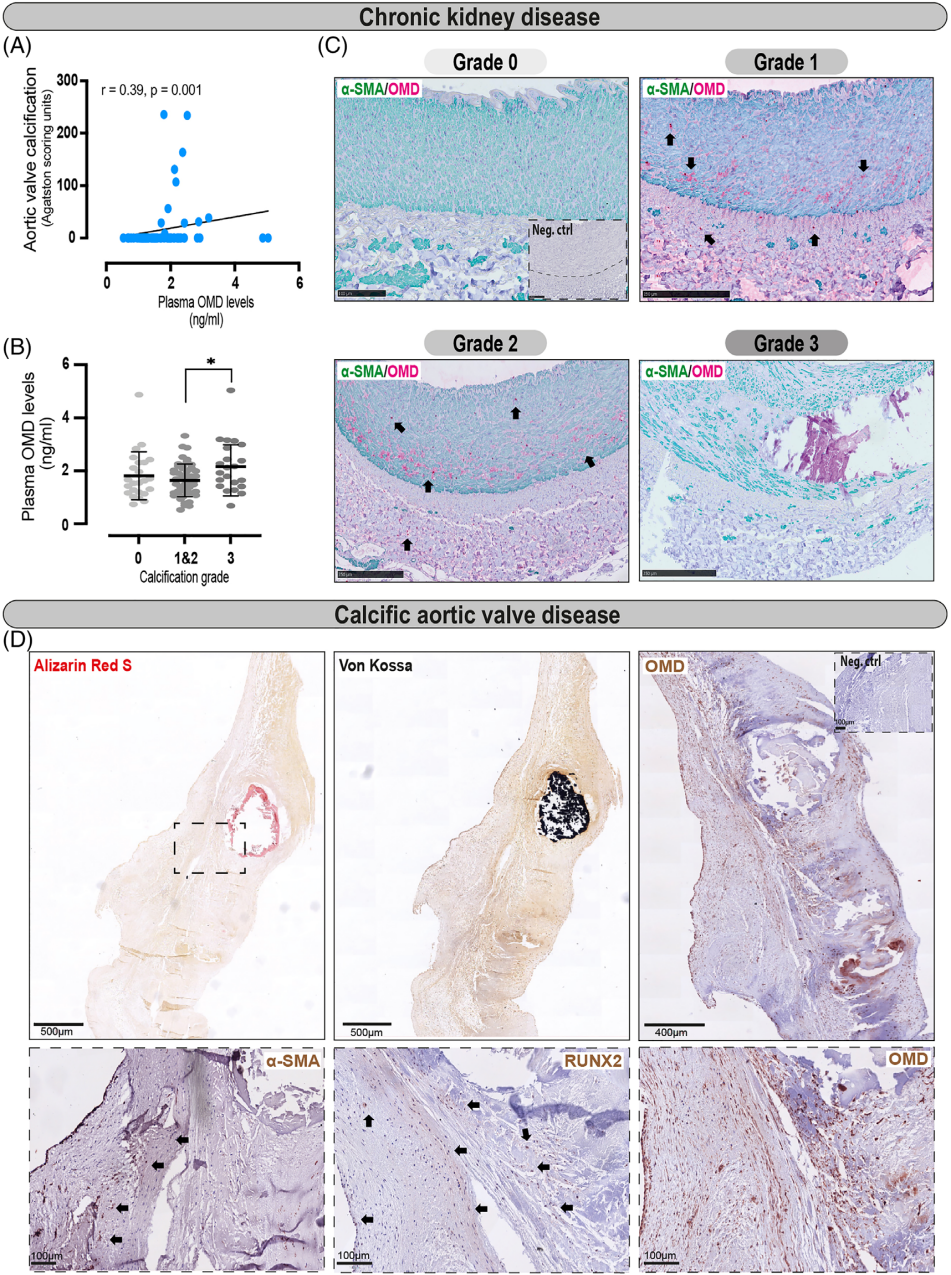
Plasma and tissue osteomodulin (OMD) protein analyses in chronic kidney disease (CKD) and calcific aortic valve disease (CAVD) patients. (A) Spearman correlation between plasma OMD levels and aortic valve calcification (in Agatston scoring units) in CKD patients (*n* = 65). (B) OMD protein measurements in plasma from CKD patients stratified in groups according to the medial calcification grade/score (CS) of epigastric arteries from these patients (ranging from 0 to 3, where 0 signifies no arterial calcification, 1 and 2 refer to moderate calcification and 3 refers to extensive arterial calcification). Number of patients per group: *n* = 25 for CS = 0, *n* = 25 for CS = 1, *n* = 24 for CS = 2, *n* = 24 for CS = 3. One‐way ANOVA multiple comparison test; data presented as mean with SD. (C) Representative histological images of epigastric arteries from CKD patients with the four different grades of calcification, immunostained for OMD (red signal) and α‐SMA (green). Arrows point to OMD positive signal in the tissues. (D) Representative images from consecutive human aortic valve leaflet slides stained with Alizarin red and von Kossa to visualise calcification, or immunostained for α‐SMA, OMD and RUNX2. Scale bar as indicated in all images. Insets show corresponding isotype negative control. Differences between groups were considered significant at *p*‐values < .05 (*p < y.05)

To evaluate the association of OMD with CKD, we first measured its levels in plasma from an existing biobank of patients undergoing living donor renal transplantation (CKD cohort, Karolinska University Hospital), along with other biomarkers of inflammation, ageing, calcification, etc. (Table 1). Demographic characteristics of the cohort are shown in Table S1. Plasma analysis (*n* = 98 individuals) showed that circulating OMD protein levels positively correlated with inflammatory markers, such as hsCRP, TNF and ageing markers like AGPs and Klotho. The analysis also showed a suggestive positive correlation of OMD levels with glucose and oxidative stress marker 8‐OHdG. A statistically significant negative correlation was found with bone turnover vitamin D metabolites (25(OH) D‐vitamin and 1,25(OH) D‐vitamin) and positive with alkaline phosphatase activity and sclerostin. While no association was observed with coronary artery calcification (CAC score), our analysis revealed a significant positive correlation with the calcification score in the aortic valves (Figure 1A).

**TABLE 1:** Univariate Spearman correlations between plasma osteomodulin (OMD) protein levels and other plasma and clinical markers in chronic kidney disease (CKD) patients related to inflammation, calcification, ageing, metabolism, bone turnover and oxidative stress

Variables (no. of patients)	Rho	*p*‐Value
Inflammation		
hsCRP, mg/L (*n* = 97)	**0.2192**	**.0338**
IL‐6, pg/ml (*n* = 50)	**0.2528**	**.0865**
TNF, pg/ml (*n* = 47)	**0.4626**	**.0016**
Calcification		
Coronary artery calcification (*n* = 67)	0.1347	.2771
Aorta valve calcification (*n* = 65)	**0.3902**	**.0013**
Ageing		
Skin AGE by autofluorescence (*n* = 79)	**0.2330**	**.0387**
Klotho, pg/ml (*n* = 52)	**0.3238**	**.0233**
Metabolic biomarkers		
Triglycerides, mmol/L (*n* = 98)	0.0332	.7493
Cholesterol, mmol/L (*n* = 98)	−0.1298	.2100
HDL cholesterol, mmol/L (*n* = 98)	0.0377	.7167
Glucose, mmol/L (*n* = 70)	**0.2214**	**.0718**
HbA1c, % (*n* = 93)	−0.0380	.7222
Haemoglobin, g/L (*n* = 74)	0.0509	.6731
TSH, mE/L (*n* = 52)	0.2312	.1099
fT3, pmol/L (*n* = 52)	−0.0526	.7198
fT4, pmol/L (*n* = 52)	−0.1817	.2114
Bone turnover		
PTH, pmol/L (*n* = 69)	−0.1551	.2033
Ca, mmol/L (*n* = 97)	0.0226	.8290
PO_4_, mmol/L (*n* = 97)	0.1172	.2607
Mg, mmol/L (*n* = 46)	0.1383	.3763
25(OH) D‐vitamin, nmol/L (*n* = 96)	**−0.2294**	**.0270**
1,25(OH) D‐vitamin, pmol/L (*n* = 58)	**−0.2733**	**.0435**
ALP, U/L (*n* = 46)	**0.3066**	**.0455**
Sclerostin, pg/ml (*n* = 50)	**0.3415**	**.0188**
Oxidative stress		
8‐OHdG, ng/ml (*n* = 43)	**0.3674**	**.0197**
Pentosidine, nmol/L (*n* = 37)	**0.3311**	**.0558**

Abbreviations: 8‐OHdG, hydroxy‐2′‐deoxyguanosine; AGE, advanced glycation end product; ALP, alkaline phosphatase; Ca, calcium; fT3, free triiodothyronine; fT4, free thyroxine; HbA1c, glycated haemoglobin; HDL, high‐density lipoprotein; hsCRP, high‐sensitivity C‐reactive protein; IL‐6, interleukin 6; Mg, magnesium; PO_4_, phosphate; PTH, parathyroid hormone; TNF, tumour necrosis factor; TSH, thyroid‐stimulating hormone.

Stratification of the measured circulating OMD protein levels into groups according to the medial calcification grade/score (CS) of epigastric arteries from these patients, showed a trend for increase of plasma OMD in patients with severely calcified arteries (Figures 1B and S1, Table S4). Immunohistochemistry revealed the presence of OMD protein intra‐ and extracellularly in both arterial media and adventitia of patients from mild‐to‐moderate calcification groups, while no signal was found in non‐calcified (CS0) or severely calcified arteries (Figure 1C).

Considering that plasma OMD levels also showed an association with the degree of aortic valve calcification in CKD patients, we explored this link using a biobank of aortic valve leaflets from patients who underwent valve replacement at MUMC+. Histological evaluation confirmed the leaflet calcification by Alizarin Red and von Kossa staining (Figure 1D). Immunohistochemistry for OMD confirmed its presence in aortic leaflets, where it was abundant around the calcified nodes and in areas with some α‐SMA^+^ and many RUNX2^+^ cells.

These data show the correlation of plasma OMD with inflammation, oxidative stress, bone turnover markers and aortic valve calcification, as well as the enrichment of tissue OMD in human medial and valvular calcification.

### 3.2 OMD levels are increased with atherosclerotic plaque calcification

**FIGURE 2:**
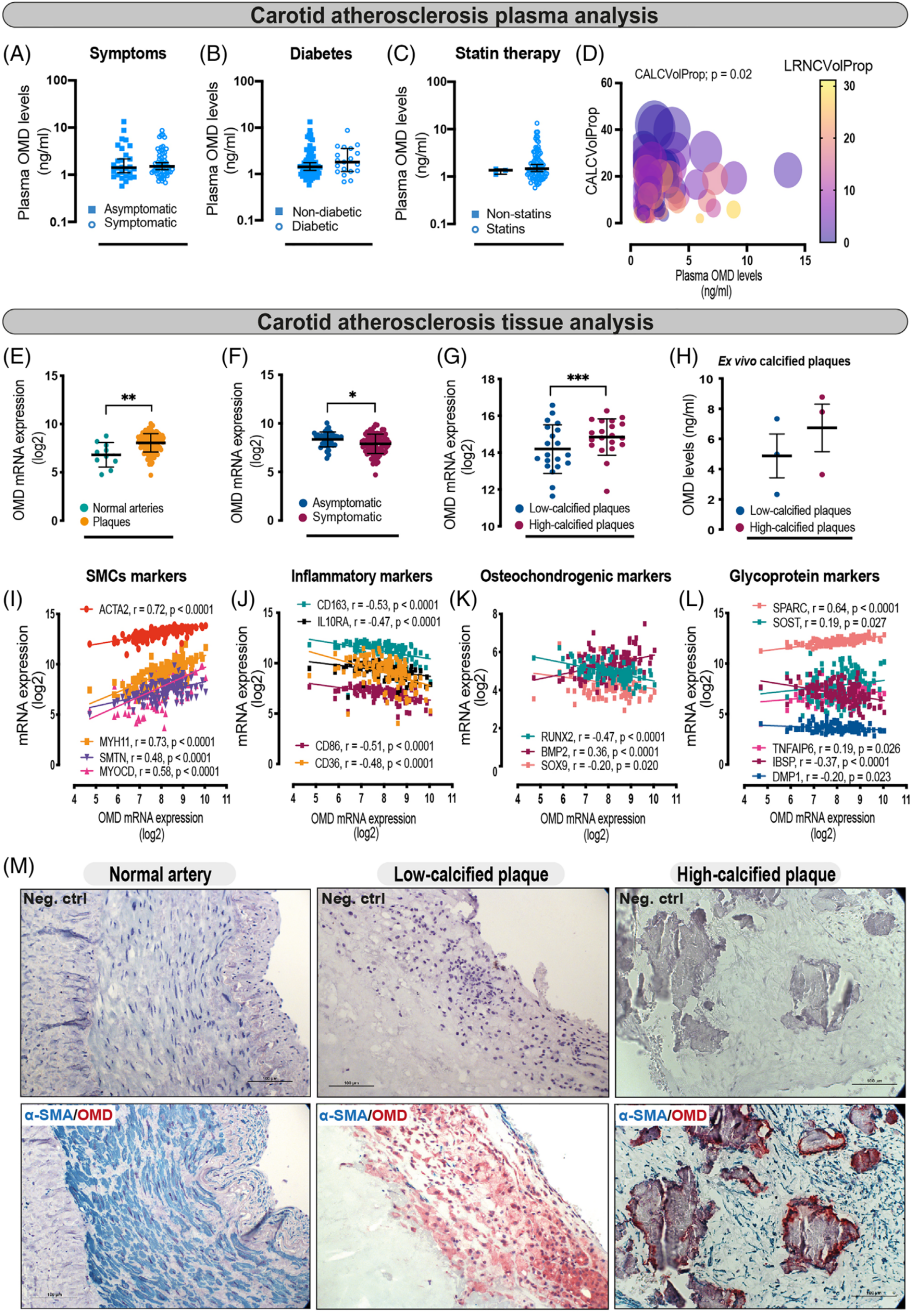
Plasma and tissue osteomodulin (OMD) mRNA and protein analyses in carotid atherosclerosis patients. (A) Plasma OMD levels compared between symptomatic (*n* = 57) and asymptomatic (*n* = 28) patients. Mann–Whitney *t*‐test; data presented as median with 95% confidence interval (CI). (B) OMD plasma protein levels stratified according to diabetic (combined type 1 and type 2; *n* = 19) and non‐diabetic individuals (*n* = 66). Mann–Whitney *t*‐test; results presented as median with 95% CI. (C) OMD protein levels in plasma from patients stratified according to lipid lowering therapy by statins. Mann–Whitney *t*‐test; data are presented as median with 95% CI. (D) Multiple linear regression analysis was used to estimate the association among plasma OMD levels and CALCVolProp (circle size), LRNCVolProp (color grade), plaque burden volume ratio and wall‐to‐lumen volume ratio as estimated by vascuCAP quantitative computed tomography (CT) image analysis software (*n* = 85). The figure is complemented by Table S5 with more detailed analysis. (E and F) OMD gene expression in microarrays from carotid atherosclerotic plaques (*n* = 127) compared to normal arteries (*n* = 10), and in plaques from symptomatic patients (*n* = 87) versus asymptomatic ones (*n* = 40). Mann–Whitney and Student's *t*‐test were performed, respectively. Data expressed as mean with SD. (G) OMD gene expression in microarrays from high‐calcified (*n* = 20) versus low‐calcified (*n* = 20) human carotid atherosclerotic plaques, where calcification was assessed by TeraRecon CT image analysis software. Student's *t*‐test, data expressed as mean with SD. (H) OMD protein measurement from the supernatants of atherosclerotic plaques (3 low vs. 3 high calcified, as estimated by vascuCAP quantitative CT image analysis software) cultured ex vivo for 24 h. Student's *t*‐test, data expressed as mean with standard error of mean (SEM). (I–L) Spearman correlations between OMD mRNA levels from tissue microarrays and the expression of typical smooth muscle cell markers, inflammatory markers, osteochondrogenic and secreted glycoprotein markers in plaques. (M) Representative images of human tissues (normal arteries and plaque specimens) immunostained for OMD (red signal) and α‐SMA (blue signal). OMD protein was not detected in normal arteries, but it was abundant in plaques, especially high‐calcified ones, where it localised to the regions rich with α‐SMA^+^ cells in the fibrous cap and around calcified nodules. Scale bar 100 μm. Differences between groups were considered significant at *p*‐values < .05 (*p < .05, **p ≤ .01, ***p ≤ .001)

To determine whether OMD shows association with end‐stage carotid atherosclerosis, peripheral plasma protein levels were measured in *n* = 85 patients from the large BiKE. Stratification of the results according to patients’ symptoms, diabetes (Figure 2A,B) or smoking (data not shown) did not reveal any significant differences, but patients receiving statin medication showed a trend for higher plasma OMD (mean ± SD of 2.294 ± 2.17 ng/ml) than those who were not (mean ± SD of 1.306 ± 0.17 ng/ml) on this therapy (Figure 2C, Table S4). To determine the association of circulating OMD with plaque's morphological characteristics, we performed multiple linear regression analysis among plasma OMD protein levels and calcification volume proportion (CALCVolProp), lipid‐rich necrotic core proportion (LRNCVolProp), plaque burden volume ratio and wall‐to‐lumen volume ratio as quantified by diagnostic carotid CT angiography scans (*n* = 85). The analysis revealed an independent statistically significant positive correlation between plasma OMD levels and plaque calcification (0.02) (Figure 2D, Table S5).

Next, we examined OMD expression in atherosclerotic plaques from these patients. OMD mRNA was upregulated in microarrays comparing plaques (*n* = 127) versus normal arteries (*n* = 10) (Figure 2E), but downregulated in plaques from S (*n* = 87) versus AS (*n* = 40) patients (Figure 2F). Interestingly, stratification of microarray data into high‐ versus low‐calcified plaques as estimated by image analysis from diagnostic carotid CT angiographies,^10^ showed that OMD transcript was significantly upregulated in high‐calcified plaques (Figure 2G). To confirm this finding, OMD protein levels were quantified with ELISA in the conditioned medium from fresh high‐ versus low‐calcified plaques maintained in ex vivo culture for 24 h, which validated a trend towards higher OMD levels released from high‐calcified plaques (Figure 2H).

To understand which cell types in plaques potentially express OMD, the transcript was correlated with various markers from microarrays. OMD correlated strongly positively with markers of typical SMCs, such as ACTA2, MYH11, SMTN and MYOCD (Figure 2I), but negatively with inflammatory markers, such as CD86, CD163, CD36, IL‐10RA (Figure 2J). Correlation of OMD with osteochondrogenic and glycoprotein markers (Figure 2K,L) revealed that the transcript was associated negatively with RUNX2, an osteogenic transcription factor essential for bone formation, and SOX9, a master regulator of cartilage differentiation, while positively with BMP2, required for osteoblast differentiation. Moreover, we found that OMD transcript was positively correlated with TNFAIP6, a potent tissue‐protective anti‐inflammatory factor, secreted protein acidic and rich in cysteine (SPARC), a calcium‐binding matricellular protein and SOST, a soluble inhibitor of canonical Wnt signalling. Immunohistochemistry showed the absence of OMD protein in normal arteries and confirmed its presence intra‐ and extracellularly in both low‐ and high‐calcified plaques, with localisation around the macro‐calcified nodules within α‐SMA^+^ areas (Figure 2M).

The data from end‐stage carotid atherosclerosis show that circulating OMD levels are increased in association with plaque calcification. Plaque tissue OMD is also enriched in association with intimal macro‐calcification and found in α‐SMA^+^ areas. Collectively, our data from human cohorts reveal the robust and specific enrichment of both vascular tissue and circulating OMD levels with calcification in patients with carotid atherosclerosis, CKD and CAVD.

### 3.3 OMD is localised around macro‐calcification nodes in murine models of medial and intimal calcification

**FIGURE 3:**
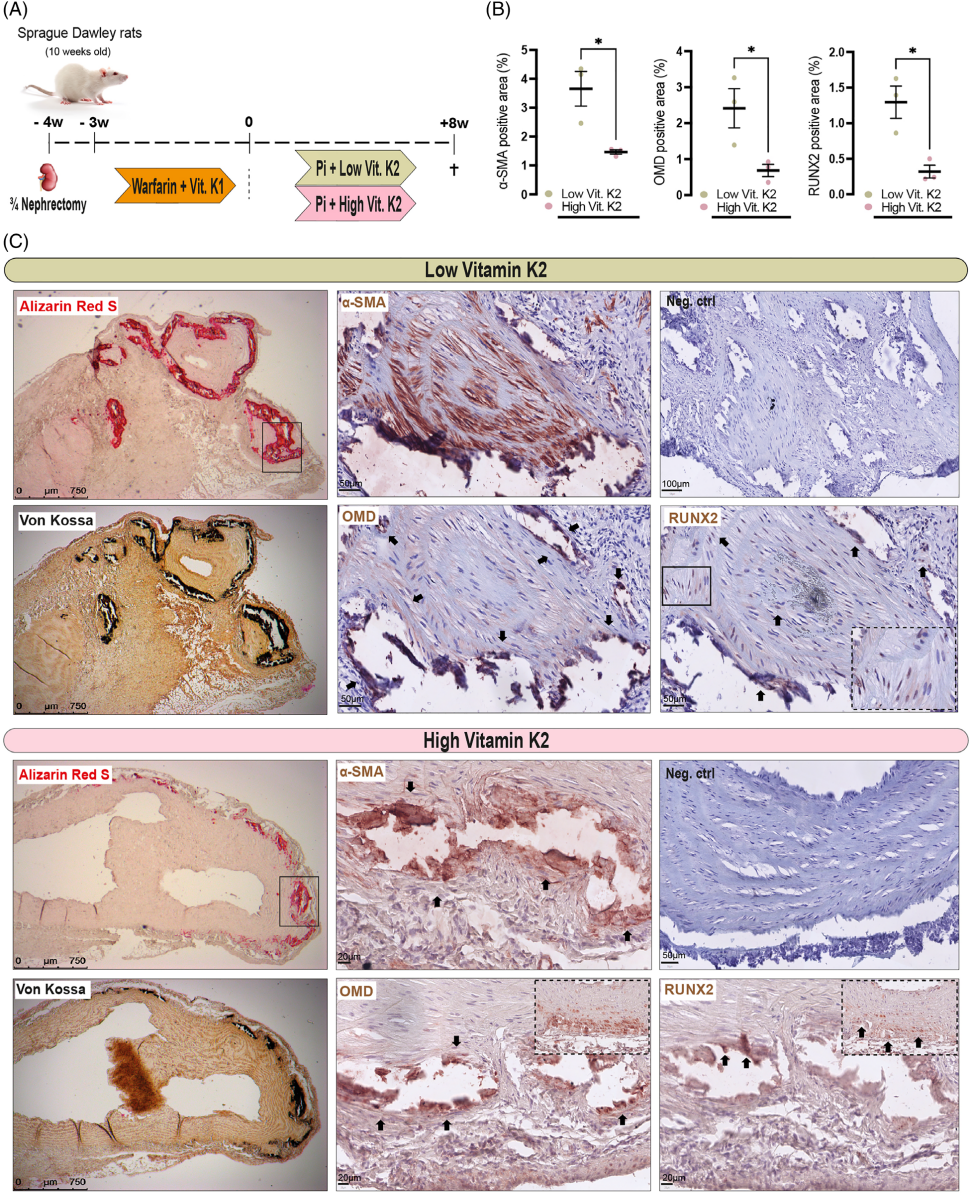
Osteomodulin (OMD) expression pattern in aortas with medial calcification from nephrectomised rats. (A) Schematic representation of the experimental procedure: 10 weeks old rats were nephrectomised and thereafter treated with 3 mg/g of warfarin and 1.5 mg/g vitamin K1 in food for 3 weeks, then switched to high phosphate (1.2%) diet with either high (100 μg) or low (5 μg) vitamin K2 for 8 weeks. (B) Percentage of positively stained areas for α‐SMA, OMD and RUNX2 in rat aortas by using ImageJ v2.0. At least three fields of view were quantified and averaged per staining per animal and at least three rats were used per condition. Statistical significance between groups was assessed by Student's *t*‐test; data expressed as mean with standard error of mean (SEM). (C) Representative histological images of aortic roots stained for Alizarin Red and von Kossa to visualise the extent of calcification, or immunostained for α‐SMA, OMD and RUNX2 markers (brown signal). Insets represent higher magnification images from the same or other regions. Arrows point to OMD signal in the extracellular matrix. Scale bar as indicated. Differences between groups were considered significant at *p*‐values < .05 (*p < .05)

To further consolidate our findings from human cohorts, we embarked on two experimental murine models. Firstly, three‐fourth nephrectomy was performed in 10 weeks old Sprague–Dawley rats. Warfarin and vitamin K1 were given to all nephrectomised rats. Next, animals received diet supplemented with high phosphate and were randomised to two groups: one group received high vitamin K2, while the other low vitamin K2 (Figure 3A). Aortic roots were collected and stained for Alizarin Red and von Kossa to visualise the resulting medial calcification resembling that of patients with CKD (Figure 3C). As expected, aortas from high vitamin K2 treated rats calcified to a lesser degree compared to the ones from low vitamin K2 treated rats.^31^, ^32^ Immunohistochemistry on consecutive slides with semi‐quantification, revealed that OMD protein was strongly expressed in aortic media from rats receiving low vitamin K2 and localised both intra‐ and extracellularly around macro‐calcified nodes (Figure 3B,C). In rats receiving high vitamin K2 and experiencing less calcified aortic media, mainly lower extracellular OMD^+^ signal was noted around calcified nodes and these areas also contained fewer α‐SMA^+^ and RUNX2^+^ cells.

**FIGURE 4:**
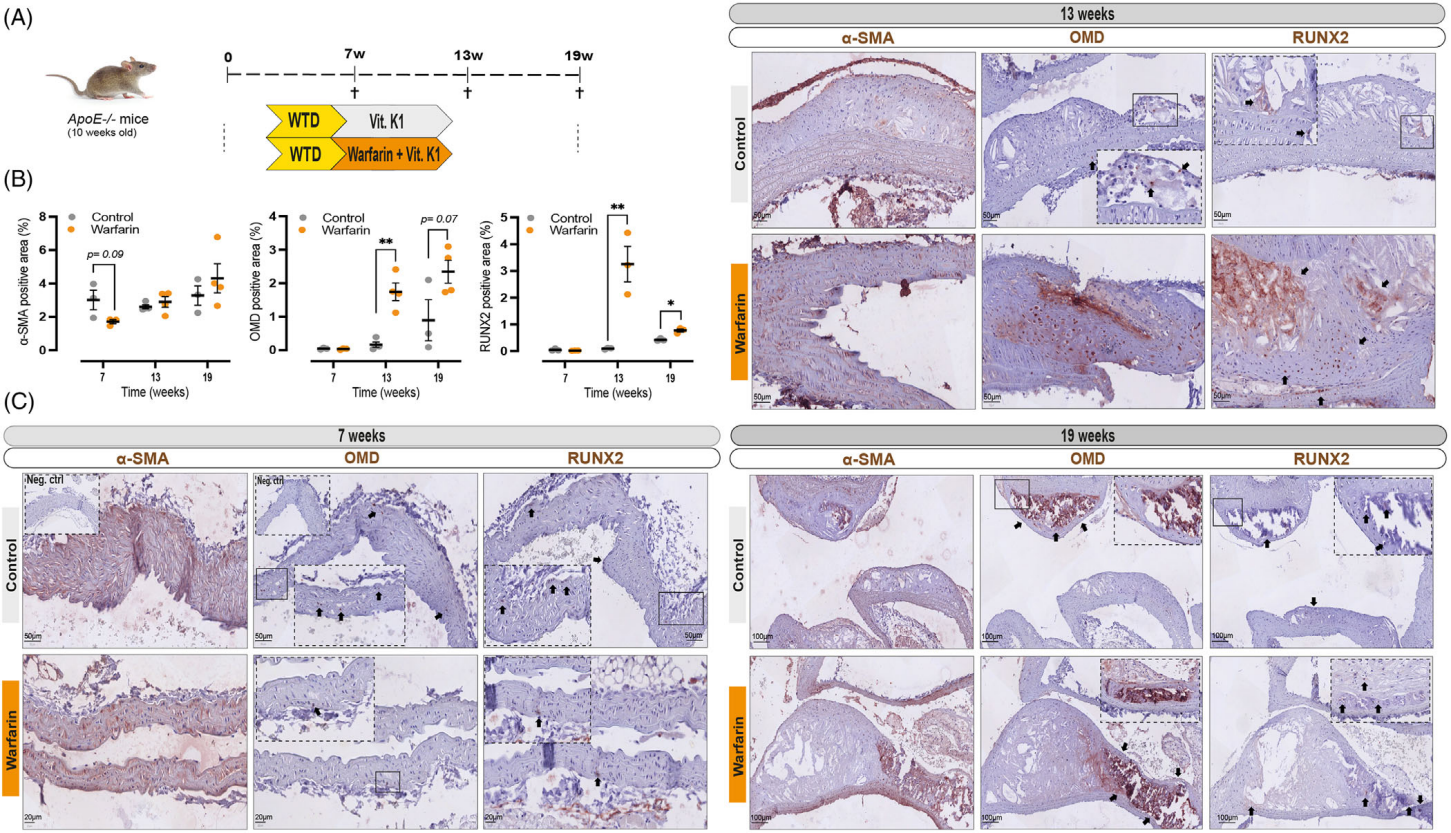
Osteomodulin (OMD) expression and localisation in aortas with intimal calcification from *ApoE*‐deficient mice. (A) Schematic representation of experimental procedure: 10 weeks old *ApoE* ^−/−^ mice received a western‐type diet (WTD) supplemented with 1.5 mg/g vitamin K1 or a WTD supplemented with 3 mg/g of warfarin and 1.5 mg/g vitamin K1 for 7, 13 or 19 weeks. (B) Percentage of positively stained areas for α‐SMA, OMD and RUNX2 in mice aortas by using ImageJ v2.0. At least three fields of view were quantified and averaged per staining per animal and at least three to four mice were used per condition. Statistical significance between groups was assessed by Student's *t*‐test; data expressed as mean with standard error of mean (SEM). (C) Representative histological images of aortas from *ApoE* ^−/−^ mice untreated (control) or treated with warfarin + vitamin K1, stained for α‐SMA, OMD and RUNX2 (brown signal). Arrows point to OMD signal. Scale bar as indicated. Insets show corresponding isotype negative control and higher magnification images from the same or other regions. Differences between groups were considered significant at *p*‐values < .05 (*p < .05, **p ≤ .01)

To address the role of OMD during atheroprogression and the development of intimal calcification, we utilised a model where *ApoE* ^−/−^ mice received a western‐type diet (WTD) supplemented with warfarin and vitamin K1^33^ (Figure 4A), gradually developing severe calcification in the aortic arch and the innominate artery over the course of 19 weeks. Histological analysis showed an abundance of OMD^+^ (both intra‐ and extracellular) and RUNX2^+^ staining in the intimal layers after 13 weeks (Figure 4B,C), while the staining was already detectable in few cells at the 7 weeks timepoint. In the more advanced 19 weeks plaques, OMD^+^ signal was mainly localised within and surrounding the ECM of the heavily calcified regions both in control and warfarin treated mice, with nearly complete absence of RUNX2^+^ signal at this late stage. It is worth noting that no OMD staining was found in the LRNC areas of these atherosclerotic lesions.

The data from these experimental models confirm that OMD protein is associated with developing medial and intimal calcification, which are processes typical for late‐stage human CKD and atherosclerosis, respectively. OMD was enriched early during the calcification process and localised in the ECM of areas abundant in α‐SMA^+^ and RUNX2^+^ cells.

### 3.4 OMD is expressed by fibromyoblasts in human atherosclerotic plaques

**FIGURE 5:**
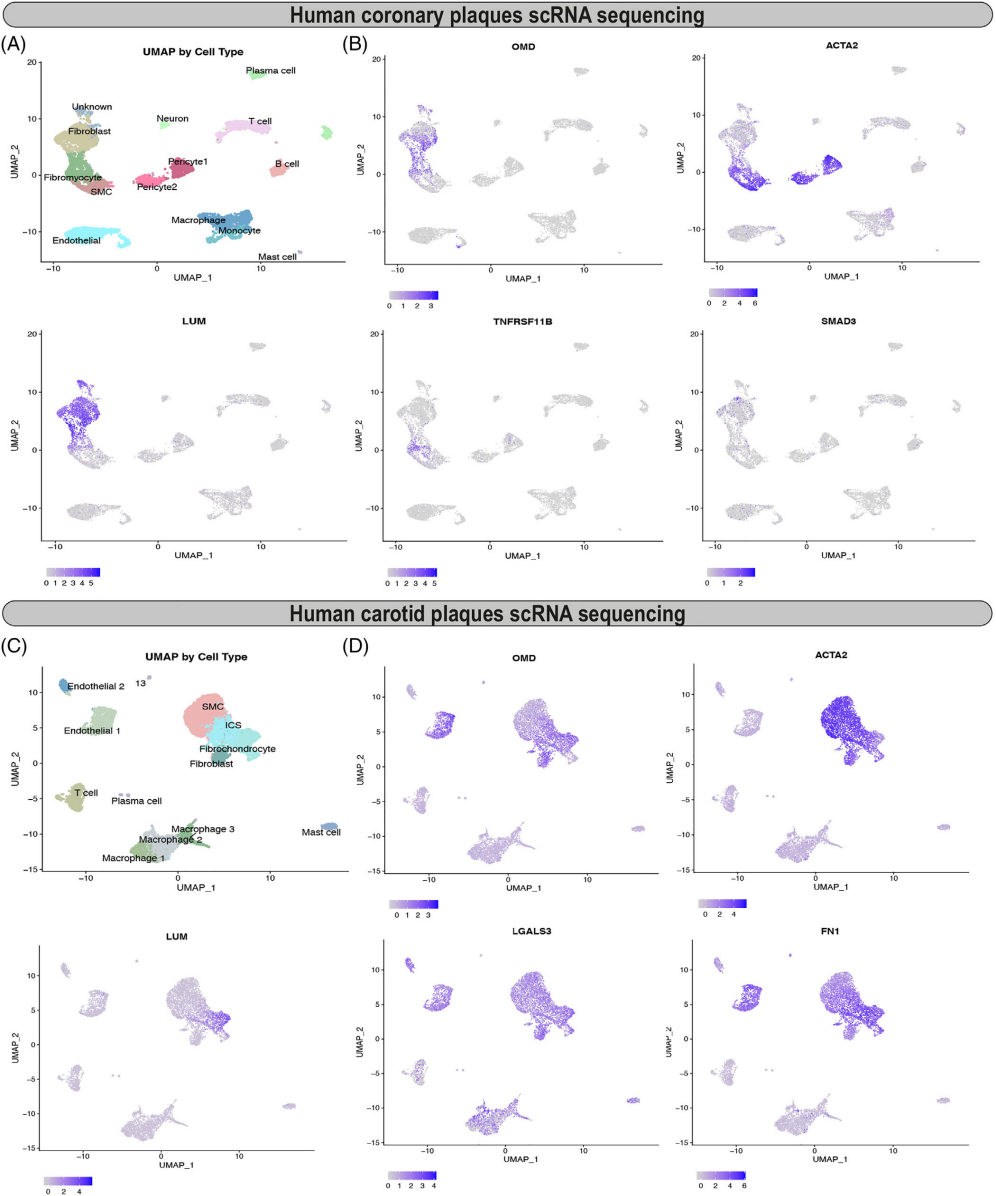
Osteomodulin (OMD) expression in cell populations in human coronary atherosclerotic plaques. (A) Uniform manifold approximation and projection (UMAP) visualisation of cell types identified in isolated human coronary atherosclerotic plaques by scRNAseq (*n* = 4 patients). (B) UMAP visualisation of cells expressing OMD, ACTA2, LUM, TNFRSF11B and SMAD3. (C) UMAP visualisation of cell types identified in isolated human carotid atherosclerotic plaques by scRNAseq (*n* = 3 patients). (D) UMAP visualisation of cells expressing OMD, ACTA2, LUM, LGALS3 and FN1

Considering that SMCs are the major cell type that undergoes phenotypic transformation during the vascular calcification process,^34^ and that OMD protein was localised within α‐SMA^+^ areas in calcified tissues, we next utilised public scRNA sequencing data of human coronary and carotid atherosclerotic plaques to investigate the association of OMD with the various ACTA2^+^ plaque cell subtypes.^35^, ^36^ In coronary plaques, bioinformatic analysis revealed that OMD was expressed by fibromyocyte and fibroblast cell fractions characterised by the gradually lower expression of ACTA2 compared to classical SMCs, but higher expression of osteoblastic markers lumican (LUM), osteoprotegerin (TNFRSF11B) and SMAD3 (Figure 5A,B). Similarly, in carotid artery plaques OMD was expressed by fibroblasts, fibrochondrocytes and multipotent intermediate cell state (ICS) fractions (Figure 5C,D) and co‐expressed with known markers of SMC phenotypic modulation, such as LUM, galectin 3 (LGALS3) and fibronectin 1 (FN1).^37^ These markers have recently been described as key signatures for the SMCs transitioning towards the fibromyoblast cell populations with multipotent features.^35^, ^36^

### 3.5 OMD is induced in SMCs by inflammatory and osteogenic cytokines, and in association with calcification

**FIGURE 6:**
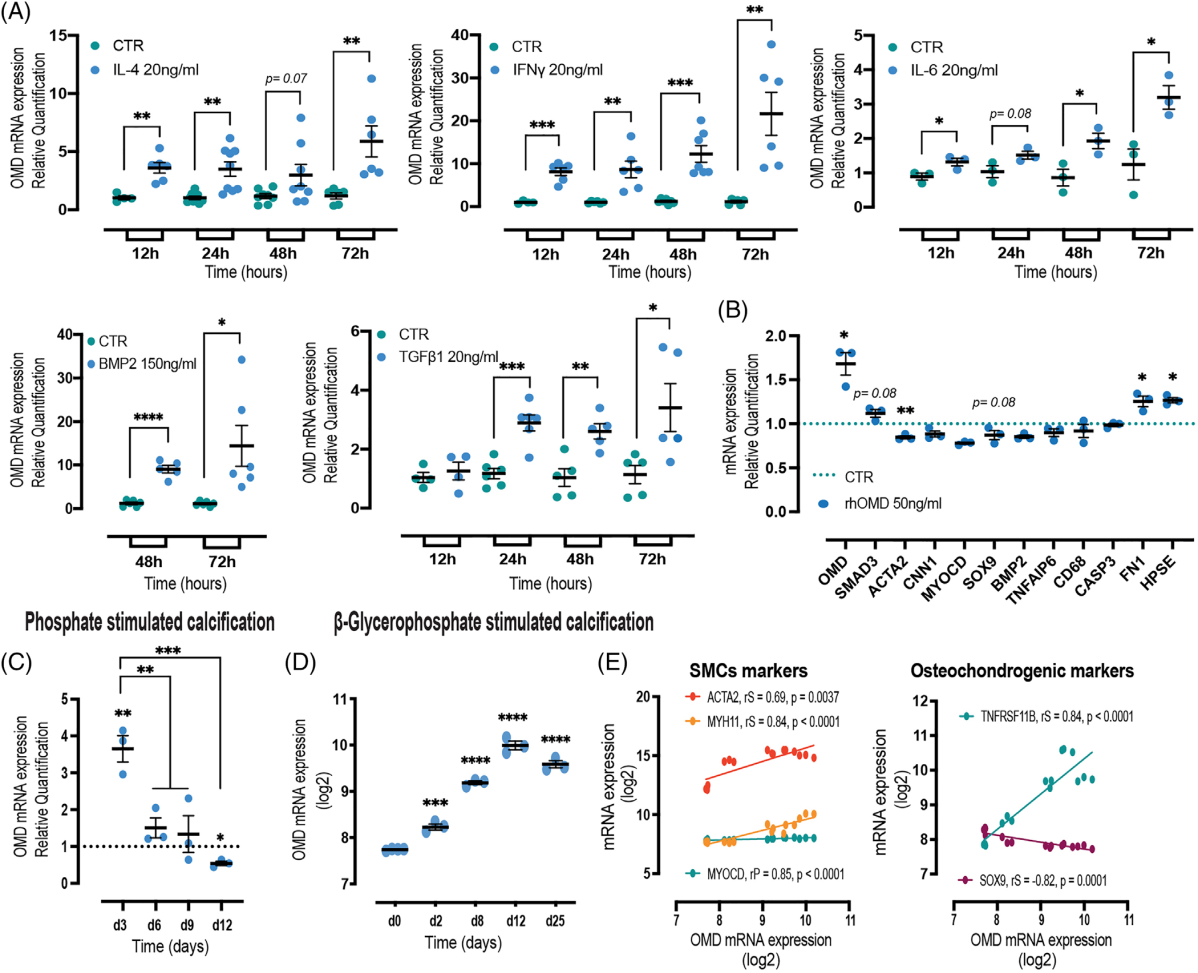
Osteomodulin (OMD) is induced in smooth muscle cells by inflammatory and osteogenic stimuli. (A) OMD mRNA expression levels in human aortic smooth muscle cells (HAoSMCs) treated with vehicle (control, CTR), IL‐4, IFNγ, IL‐6, BMP2 or TGFβ1 for 12, 24, 48 or 72 h. An increase of OMD gene expression was detected only after 48 h of BMP2 treatment. The experiment was performed in triplicate. Statistical significance between groups was assessed by Student's *t*‐test; data expressed as mean with standard error of mean (SEM). (B) Gene expression analysis of typical smooth muscle cell markers, osteochondrogenic, inflammatory markers of HAoSMCs treated with 50 ng/ml human recombinant OMD (rhOMD) for 24 h. The values for genes of interest in rhOMD treated HAoSMCs were normalised with the corresponding values of non‐treated control cells (green dotted line). Statistical significance between groups was assessed by Student's *t*‐test; data expressed as mean with SEM. (C) OMD mRNA expression levels in HAoSMCs treated with 2.6 mM Pi for up to 12 days. The experiment was performed in triplicate with cells from human biopsies. Statistical significance between groups was assessed by Student's *t*‐test and one‐way ANOVA multiple comparison test; data expressed as mean with SEM. (D) OMD mRNA expression levels in human coronary smooth muscle cells (HCoSMCs) treated with osteogenic medium consisting of 0.1 mM ascorbic acid, 10 mM β‐glycerophosphate and 100 nM dexamethasone, for promoting the osteoblast phenotype. Statistical significance between groups was assessed by one‐way ANOVA multiple comparison test; data expressed as mean with SEM. (E) Correlations between OMD mRNA levels and the expression of typical smooth muscle cell markers (left) and osteochondrogenic markers in the same cells (right). Differences between groups were considered significant at *p*‐values < .05 (*p < .05, **p ≤ .01, ***p ≤ .001, ****p ≤ .0001). Data in (D and E) were extracted from Alves et al. public microarray dataset (GEO accession no. GSE37558)

To mechanistically test the causative role of OMD in promoting SMC‐to‐fibromyoblast transition, we performed further investigations in primary human aortic smooth muscle cells (HAoSMCs) in vitro. We found that OMD mRNA levels were increased in HAoSMCs after exposure to both pro‐inflammatory (IL‐4, IL‐6, IFNγ) and pro‐osteogenic (BMP2 and TGFβ1) stimuli (Figure 6A). In particular, IFNγ and BMP2 strongly potentiated OMD expression already after 12 and 48 h, respectively (up to 40‐fold). Exogenous administration of recombinant human OMD (rhOMD) for 24 h also induced upregulation in mRNA levels of OMD and SMAD3, as well as the ECM protein FN1 and heparinase (HPSE; involved in proteoglycan metabolism), whereas downregulation in mRNA levels of ACTA2 and osteochondrogenic SOX9 was observed. Overall, there were no significant changes in the gene expression of other typical SMC markers (CNN1, MYOCD), osteogenic BMP2 and inflammatory/apoptotic (TNFAIP6, CD68, CASP3) markers compared to baseline (Figure 6B).

In order to characterise the functional role of extracellular OMD on SMCs, exogenous rhOMD was added to HAoSMCs in vitro and the effects evaluated in wound healing and proliferation assays. We found that rhOMD delayed SMC migration after 24 h (Figure S2A), although it increased proliferation rate after 16 h (Figure S2B), showing that exogenous OMD has the capacity to induce SMC activation.

Then, we investigated the impact of calcification on OMD gene expression by stimulation of HAoSMCs with 2.6 mM Pi (Figure 6C) for up to 12 days. While high Ca did not induce OMD expression, high Pi treatment induced an upregulation of OMD already after 3 days, which gradually normalised to baseline levels. Bioinformatic analysis of a publicly available dataset,^38^ in which treatment of human coronary smooth muscle cells (HCoSMCs) with osteogenic medium (ascorbic acid, β‐glycerophosphate and dexamethasone) gradually led to their osteoblastic transdifferentiation, showed a strong time‐dependent upregulation of OMD mRNA levels already from day 2 with peak after 12 days of stimulation (Figure 6D). Indeed, similar experiments conducted by us, validated these results (Figure S3A). In these cells, OMD was positively correlated to typical SMC markers and markers of osteoblastic transformation (TNFRSF11B) while inversely to the chondrogenic marker SOX9 (Figure 6E).

### 3.6 Extracellular OMD inhibits extracellular matrix calcification by attenuating SMC osteoblastic transition

**FIGURE 7:**
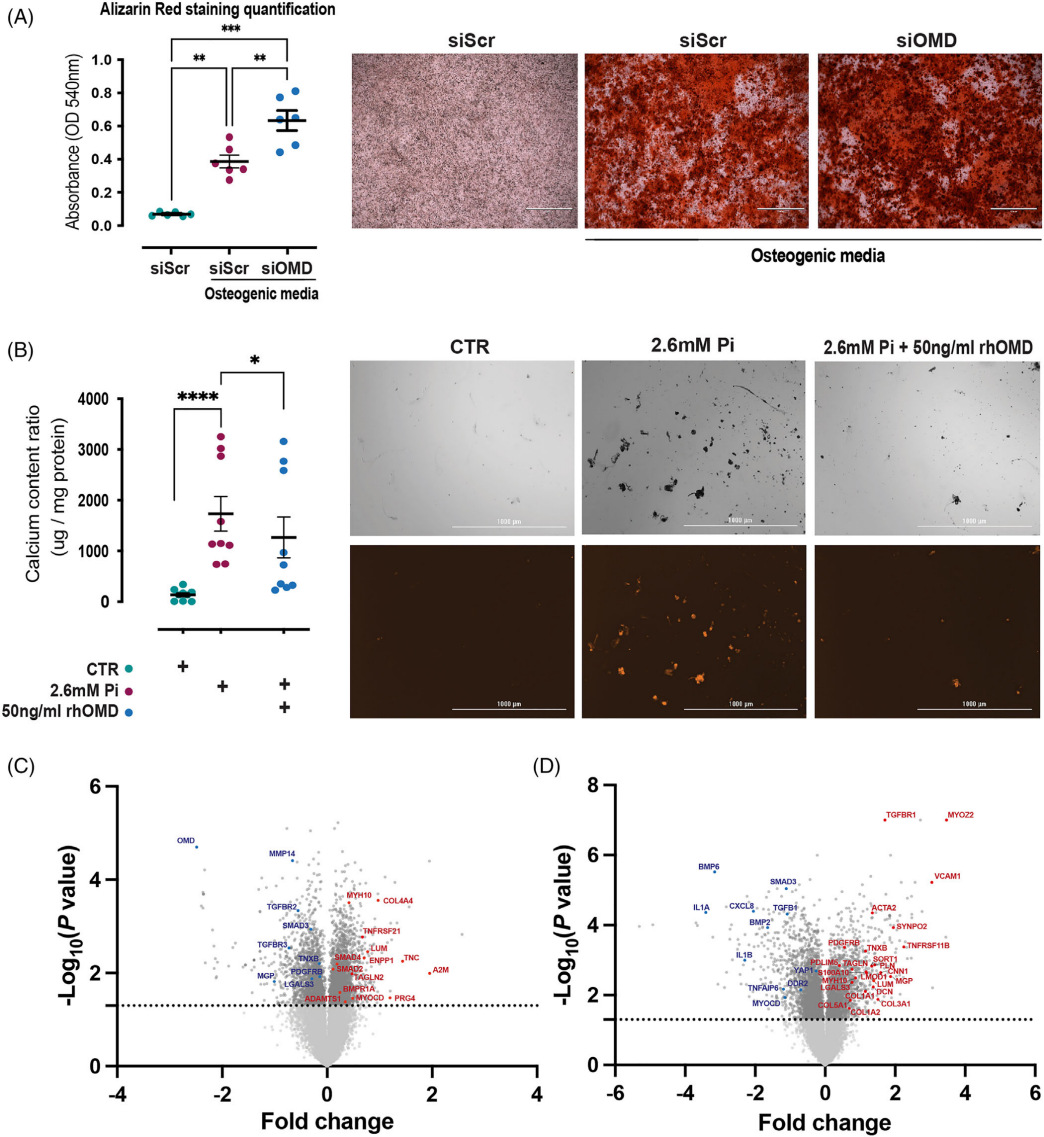
Extracellular osteomodulin (OMD) attenuates smooth muscle cell osteogenic transition and represses calcification. (A) Quantification of the in vitro calcification of human coronary smooth muscle cells (HCoSMCs) treated with siRNA for OMD or scramble control in osteogenic medium consisting of 0.1 mM l‐ascorbate phosphate, 10 mM β‐glycerophosphate and 10 nM dexamethasone for 14 days (*n* = 3 independent experiments in duplicates). Statistical significance between groups was assessed by one‐way ANOVA multiple comparison test; data expressed as mean with standard error of mean (SEM). Representative images of the calcification assay as it was visualised by Alizarin Red staining. (B) Quantification of the in vitro calcification of human aortic smooth muscle cells (HAoSMCs) treated with 2.6 mM Pi for 12 days in the absence or presence of 50 ng/ml human recombinant OMD (rhOMD) protein. The experiment was performed in triplicate with cells from human biopsies. Statistical significance between groups was assessed by one‐way ANOVA multiple comparison test; data expressed as mean with SEM. Representative images of the calcification assay where calcification was visualised by an Alexa Fluor 546 coupled fetuin A probe. Scale bar 1000 μm. (C) Volcano plot showing the top significantly upregulated (red) and downregulated (blue) genes comparing HCoSMCs treated with siRNA for OMD (*n* = 3) versus scramble control (*n* = 3) in osteogenic medium for 14 days. (D) Volcano plot showing the top significantly upregulated (red) and downregulated (blue) genes comparing HAoSMCs treated with rhOMD (*n* = 3) versus control (*n* = 3) in osteogenic medium for 6 days. Differences between groups were considered significant at *p*‐values < .05 (*p < .05, **p ≤ .01, ***p ≤ .001, ****p ≤ .0001)

Since OMD could be induced in early stages of SMC osteoblastic transition by osteogenic medium (β‐glycerophosphate), we employed RNA silencing of OMD gene expression in HCoSMCs under this condition to assess the calcification formation. The results showed that OMD silencing significantly increased ECM calcification compared to the scramble control (Figure 7A). To address the molecular and functional characteristics of this state, we performed global analysis of the differentially expressed genes between siOMD treated cells and scramble controls in osteogenic medium for 14 days. As expected, OMD mRNA levels were repressed (Figures 7C and S3B), but classical SMC markers (MYH10, MYOCD, TAGLN2) were upregulated in siOMD condition. Consistent with increased calcification, osteogenesis‐related genes (SMAD2, SMAD4, BMPR1A, ENPP1, TNFRSF21) as well as ECM mineralisation‐related genes (A2M, PRG4, TNC, LUM, COL4A4, ADAMTS1) were all increased in siOMD cells. Conversely, MMP14 gene needed for SMC migration was downregulated, along with the inflammatory modulation marker LGALS3, de‐differentiation marker PDGFRB and calcification inhibitor MGP. The TGFβ signalling pathway molecules (TGFBR2, TGFBR3, SMAD3) were downregulated in siOMD cells as well. Interestingly, silencing of SMAD3 repressed OMD expression, while promoting early osteoblast BMP2 gene and ECM molecules COL4A4, COL10A1 and CDH2 (Figure S4).

Next, we tested the effects of exogenous OMD on the development of calcification nodules in vitro. Indeed, addition of full length rhOMD to HAoSMCs in the 2.6 mM Pi culture media for 12 days, reduced the ECM mineralisation and calcified nodules formation (Figures 7B and S5), suggesting that extracellular OMD may have a protective effect in attenuating calcification. To interrogate the mechanism, we again performed global gene expression analysis of HAoSMCs treated with or without rhOMD in high Pi medium for 6 days. The analysis revealed that rhOMD induced contractile (ACTA2, CNN1, TAGLN, SYNPO2, PLN, LMOD1, PDLIM5) markers of typical SMCs,^29^ but also MYH10, TNFRSF11B, LGALS3, S100A10, SORT1 and VCAM1 which mark the more de‐differentiated fibroblast‐like SMCs in the fibrous cap (Figure 7D). In addition, markers of SMC myogenic differentiation such as MYOZ2, TGFBR1 and PDGFRB were upregulated in rhOMD cells, while TGFβ1 and MYOCD, a transcription factor promoting the SMC lineage, were markedly downregulated. Moreover, these cells exhibited ECM secretory capacities as depicted by the striking upregulation of LUM, DCN, various collagens as well as the increased expression of the calcification inhibitor MGP. Conversely, the pro‐inflammatory cytokines IL1A, IL1B, CXCL8, TNFAIP6, the osteoblastic markers BMP2, BMP6, SMAD3, DDR2 and the matrix stiffness sensing marker YAP1 were significantly downregulated in rhOMD treated SMCs treated in high Pi medium. Gene expression analysis by real‐time quantitative polymerase chain reaction (RT‐qPCR) confirmed the above findings and revealed that rhOMD also prevented the upregulation of osteochondrogenic markers (SMAD3, BMP2, similar trend observed for SOX9) and inflammatory markers (CD68, TNFAIP6), without affecting cell apoptosis (CASP3) (Figure S6A). At protein level, rhOMD repressed IL‐1β secretion from SMCs to control levels compared to Pi only at day 6. However, this effect was gradually attenuated along with the progression of calcification (Figure S7). Consistent with differentially expressed genes in response to rhOMD, ECM organisation, proteoglycans and glycosaminoglycans biosynthesis, collagen and elastic fiber formation, cell–matrix interactions, smooth muscle contraction and retinoid acid biosynthesis were among the top biological pathways identified in enrichment analysis of upregulated genes (Figure S8). TGFβ, BMP, Wnt, NOTCH signalling pathways related to osteoblastic transition and calcification were repressed, along with pathways involved to innate immunity and cellular senescence. These integrative analyses suggest that rhOMD in osteogenic medium maintains SMCs contractility by delaying the cell transition towards an osteoblastic phenotype and attenuating ECM calcification, while it induces SMC reprogramming into a protective secretory myofibroblast‐like state. Of note, the protective effects of extracellular rhOMD on SMC modulation were lost after 12 days of exposure (Figure S6B).

### 3.7 The mechanistic link among OMD, SMAD3 and BMP2 in reprogramming of SMC phenotype

It was recently reported that BMP2, involved in vascular calcification by enhancing osteoblast‐like differentiation of vascular SMCs,^39^ may be linked with OMD as a positive regulator of osteogenesis through BMP2 signalling in bones.^22^ We examined whether BMP2 alone or in combination with rhOMD could be involved in osteoblast differentiation of SMC and matrix calcification after 6 days of treatment. Exogenous administration of BMP2 alone or in combination with rhOMD induced upregulation in mRNA levels of typical SMC markers as well as SMAD3, SP7 and ALPL. BMP2 downregulated osteochondrogenic (SOX9, BMP2) markers, but highly increased OMD mRNA expression, while the combination of BMP2 and rhOMD downregulated only BMP2 gene expression levels (Figure S9A). The same results were observed when SMCs were treated in osteogenic high Pi sources (Figure S9B), where both BMP2 and the combination treatment increased the osteoblastic SP7 and ALPL markers. Of note, BMP2 alone was not sufficient to induce ECM mineralisation at day 6, whereas the combination treatment increased ECM mineralisation to the same extent as high Pi conditions alone (Figure S9C).

**FIGURE 8:**
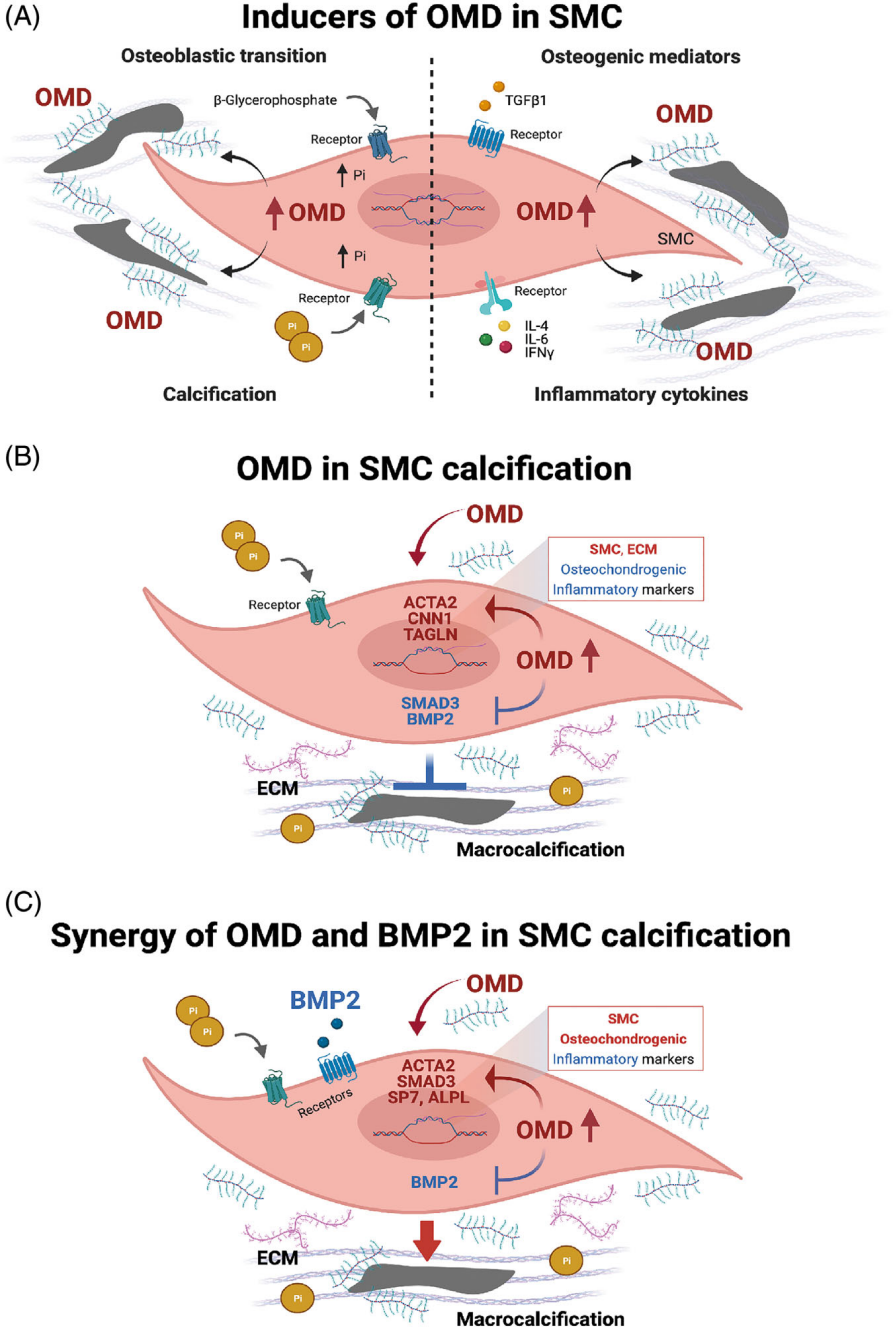
Schematic representation of the mechanism by which osteomodulin (OMD) could mediate macro‐calcification formation by smooth muscle cells

Together, our in vitro studies show that pro‐inflammatory and pro‐osteogenic cytokines, as well as calcifying and osteogenic medium enriched with Pi, are all potent and rapid inducers of OMD in SMCs (Figure 8A). Mechanistically, exogenous OMD attenuates SMC calcification by slowing down the SMC osteoblastic transition via SMAD3, while concomitantly stimulating even higher expression of intracellular OMD (Figure 8B). However, in synergy with BMP2, extracellular OMD promotes SMC modulation towards osteoblastic differentiation and ECM calcification (Figure 8C).

## 4 DISCUSSION

In this study, we present the first evidence for the role of OMD in cardiovascular calcification with several novel findings. We show that: (1) plasma OMD levels correlate with biomarkers reflecting inflammation, oxidative stress and bone turnover in CKD patients. The protein is found in mild‐to‐moderately calcified patient arteries and vessels of nephrectomised rats. (2) Circulating OMD levels positively correlate with cardiac valvular calcification and an abundance of OMD protein is present in calcified valve leaflets of patients suffering from aortic stenosis. (3) OMD plasma levels are increased in correlation with atherosclerotic plaque calcification and OMD is abundantly present in macro‐calcified carotid plaques from patients as well as in *ApoE* ^−/−^ mice. (4) In human plaques, OMD is associated specifically with SMCs transitioning to a fibromyoblast phenotype characterised by expression of osteoblast markers. (5) Finally, OMD is produced by SMCs exposed to pro‐inflammatory and pro‐osteogenic stimuli (i.e. high Pi sources). Extracellular OMD attenuates SMC calcification by slowing down their osteoblastic transition via SMAD3 signalling, but also partially by increasing the intracellular production of OMD as a feedback loop, whereas its combination with BMP2 positively regulates ECM mineralisation. Taken together, this study identified OMD as a novel circulatory and tissue factor involved in modulation of cardiovascular calcification.

As plasma OMD has been reported to associate with cardiovascular risk traits and type 2 diabetes,^16^, ^17^ we first assessed circulating levels of OMD in CKD stage 5 patients, a group with documented rapid development of early vascular ageing (EVA).^40^ In this high‐risk group of patients, EVA is characterised by the increase of inflammatory markers and metabolic dysregulation caused by oxidative stress and biochemical bone turnover abnormalities, resulting in calcification of vascular and soft tissues.^41^ The association between OMD and hsCRP and TNF is interesting as these markers are strong predictors of CVD mortality and mediate atherogenesis.^42^, ^43^ Osteolytic and bone demineralisation processes that take place during ageing may explain the negative correlation of plasma OMD levels with vitamin D.^42^ In addition, the positive correlation of circulating OMD levels with klotho^44^ and sclerostin,^23^ both strong inhibitors of calcification,^45^, ^46^ implied a possible protective function of OMD in the mineralisation process. Numerous studies have suggested that klotho is critical for vascular health and its therapeutic administration in CKD can exert vasculo‐protective effects.^44^ A recent meta‐analysis revealed a significant association between decreased soluble klotho level and increased risk of vascular calcification in CKD patients, raising the possibility of applying soluble klotho as a calcification biomarker in CKD populations.^47^ In our study, stratification of patients according to the extent of arterial medial calcification, revealed an enrichment of circulating OMD in the advanced calcification group, nevertheless the cohort was too small to study the association with klotho more specifically. However, whereas the OMD protein was present in the media and adventitia of arteries with mild‐to‐moderate calcification, it was not expressed in arteries with severe calcification. A plausible explanation could be that the presence of OMD protein in the tissue reflect ongoing arterial inflammation, oxidative stress and calcification processes that occur at the early (but not late) stages of CKD. The presence of positive intra‐ and extracellular immunostaining for OMD protein in arteries without obvious macro‐calcifications suggests that its expression and deposition might precede the advanced medial calcification. Severe medial calcification is actually characterised by the absence of inflammatory cells, thus the lack of inflammatory triggers could explain the repression of tissue OMD signal at the late stage.^48^

Abnormal metabolism of Pi in the uremic milieu contribute to the development of vascular damage and promote transdifferentiation of SMCs towards an osteoblast‐like phenotype, deposition of matrix proteins and subsequent ECM mineralisation along with a late‐stage repression of inflammatory processes.^48^ To further study tissue expression of OMD in the context of abnormal mineral metabolism, a CKD rat model, where partial nephrectomy was coupled with high Pi, warfarin and vitamin K treatment, was employed to accelerate arterial calcification in the uremic milieu.^49^, ^50^ Vitamin K2‐dependent proteins initiate a preventive mechanism for the development of vascular calcification.^31^, ^32^ Our findings confirm that aortas from rats treated with high vitamin K2 calcified less compared to those treated with low vitamin K2. This was linked with OMD protein expression that was mainly extracellular and restricted around the calcified nodes. In contrast, aortas from low vitamin K2 treated rats presented an abundance of both intra‐ and extracellular OMD, localised in areas with osteoblastic activity rich with α‐SMA^+^ and RUNX2^+^ cells.

Interestingly, we also found that circulating OMD levels correlated with the degree of calcification of aortic valve leaflets in CKD5 patients and that the protein was abundantly present in calcified leaflets from non‐renal patients who underwent aortic valve replacement. Valvular interstitial cells (VICs) positive for α‐SMA are the main structural cells involved in pathological processes associated with leaflet fibrosis, collagen fiber disorganisation and calcification.^51^ Similar to vascular SMCs, valvular calcification is underlined by osteoblastic transition of VICs as a central step orchestrated by pro‐inflammatory cytokines, towards reaching an osteogenic phenotype.^6^, ^52^ TGFβ and BMP signalling pathways, known regulators of vascular SMC calcification and osteoblastic differentiation, have been found to localise in calcified aortic valves,^53^ ^—^ ^55^ where TGFβ1 and BMP2 are expressed by myofibroblasts and pre‐osteoblasts^56^ and induce VICs pro‐osteogenic activation.^57^ Moreover, OMD has been described as a mechanosensitive gene,^58^ enriched in regions exposed to high mechanical forces such as the cardiac valves.^59^ Thus, it is not surprising that we found OMD protein broadly in the valve leaflets and especially in proximity of large‐calcified nodules, again colocalising in the same areas with α‐SMA^+^ and RUNX2^+^ cells.

In patients with carotid atherosclerosis, plasma OMD levels were not significantly changed in groups stratified according to symptoms, comorbidities and medication. The lack of association between plasma OMD levels and diabetes, validated previous reports where plasma OMD was decreased only in diabetic patients without coronary artery disease.^17^ However, extending the clinical data from CKD and CAVD patients, circulating OMD levels in carotid atherosclerotic patients strongly positively correlated to plaque calcification. Tissue OMD was increased in plaques versus control arteries, but it was downregulated in plaques from S versus AS patients, suggesting again that OMD could have a protective role. In plaques, the transcript correlated positively to typical markers of SMCs and with the osteoblastic marker BMP2, while negatively to markers of inflammatory cells. These correlations could partially be explained by the severe plaque heterogeneity in late stages of the disease characterised by macro‐calcifications, along with suppressed inflammatory pathways.^10^ We also found a negative correlation of tissue OMD with pro‐calcifying DMP1 and IBSP glycoproteins, but positive with sclerostin (SOST, as in plasma from CKD patients) and osteonectin (SPARC) that presents strong binding affinity for type I collagen and hydroxyapatite,^60^ similarly as previously reported for OMD.^61^ The direct link of tissue OMD with calcification was confirmed by its upregulation in plaques stratified into high‐ versus low‐calcified according to pre‐operative CT angiography images. Immunohistochemistry validated these findings, showing that OMD was completely absent from control vessels but enriched in plaques, where intra‐ and extracellular signal for OMD was found in the fibrous cap of low‐calcified plaques, whereas in high‐calcified plaques OMD was present closely around the calcification nodes in regions with α‐SMA^+^ cells.

This finding was further explored in a longitudinal atherosclerotic mouse model where OMD was found to be expressed intra‐ and extracellularly from early stages of atheroprogression, specifically at sites of intimal calcification. OMD^+^ staining was again present in regions abundant with α‐SMA^+^ and RUNX2^+^ cells, but completely absent from lipid‐rich regions of the necrotic core. Similar expression pattern and specific accumulation of OMD towards the mineralisation areas has been previously described in tooth development.^20^ Taken together, our results from human cohort studies and murine models highlight that OMD can be induced by inflammatory milieu, which is typical for early stages of vascular diseases, and appears to be generated mainly in the α‐SMA^+^ regions of the arterial wall and aortic valve leaflets with osteoblastic activity.

Recent single‐cell sequencing studies have convincingly shown that SMCs undergoing phenotypic modulation in atherosclerotic plaques exhibit a shift in gene expression along a continuous trajectory from a contractile SMC towards a fibroblast‐like cell, termed “fibromyocyte^35^, ^36^”. This shift is characterised by a decreasing gradient of typical SMC gene expression and an increase in markers of osteoblasts.^36^ We were able to map OMD to the same cell population, which we show is also coupled to an increase in OMD expression, possibly with the purpose to provide an adhesive matrix (e.g. FN, LUM)^62^ for later stages of the mineralisation process and macro‐calcification formation.

This notion was confirmed in vitro, where we showed that OMD could be upregulated in SMCs by different pro‐inflammatory and pro‐osteogenic stimuli. In agreement with positive correlations from plaques and plasma, IFNγ, TGFβ1 and especially BMP2 cytokines (known to play a role in modulating the cellular osteoblastic differentiation), had a potent and rapid stimulatory effect on OMD gene expression in SMCs. Similarly, exogenous administration of OMD protein solely was sufficient to activate SMCs into a synthetic phenotype, inducing endogenous OMD expression and ECM reorganisation, possibly via SMAD3 transcription factor, while generally preventing other changes in expression of SMC markers and markers of osteoblastic or inflammatory transition. To this end, OMD was also upregulated after exposure to high Pi and osteogenic media, strong drivers of osteoblastic transdifferentiation of SMCs,^63^ suggesting that OMD could serve as a mechanosensitive marker in reaction to vascular ECM changes. In addition, silencing of OMD gene expression modulated SMC phenotype by inducing the osteogenesis‐related genes and therefore, enhancing the ECM calcification. Conversely, exposure to exogenous OMD protein under calcifying conditions reprogrammed SMCs towards an intermediate fibromyoblastic state, maintaining their contractile and ECM secretory phenotype,^64^ while at the same time inhibiting the pro‐inflammatory IL‐1β cytokine secretion. Overall, this resulted in strongly attenuated ECM calcification, by slower SMC osteoblastic transition. Although these effects were lost at late stages of the calcification process, likely because SMCs activate other mechanisms to bypass the protective effects of OMD, evidence shows that OMD actively orchestrates the ECM remodelling process at earlier stages. Moreover, vascular calcification recapitulates many features of endochondral ossification, where BMP2 and OMD were recently shown to directly interact.^22^ In our study, exogenous BMP2 alone induced osteoblastic reprograming of the cells without yielding significant ECM mineralisation. It is interesting that it also downregulated endogenous BMP2 synthesis, but highly increased OMD mRNA expression. Exogenous administration of OMD together with BMP2 in SMCs under high Pi sources, resulted in calcification to the same extent as Pi alone, suggesting that OMD by interacting with BMP2 may in fact override the effects of OMD and BMP2 alone and positively regulate the mineralisation process. Our results indicate that OMD interaction with BMP2 exerts synergistic effects not only in bone^22^ but also in vascular biology.

### 4.1 Limitations

Our study represents the first comprehensive investigation of the OMD role in cardiovascular calcification, however, several limitations should be considered. As BiKE, CKD and CAVD cohorts comprise heterogeneous and metabolically highly inflamed patients with only advanced disease state and late‐stage lesions, role of OMD in disease initiation and progression could not be studied. A more detailed examination of OMD in non‐calcified valves as well as in plasma from these patients was hampered by the lack of material in this biobank. In addition, we acknowledge that inclusion of in vivo animal experiments in order to confirm the functional role of OMD in cardiovascular calcification would have considerably improved the study, however it was not feasible. To address the SMC behaviour in different vascular beds relevant for this study, we have used SMCs of different origin and found that they behaved similar with respect to the expression of typical markers and transdifferentiation properties in our experiments. Furthermore, primary human aortic SMCs at low and high passages were used in this study. While these cells express the typical markers and have functional features of differentiated SMCs, we cannot exclude that some of the more sensitive markers are already downregulated even at the early stage after cell isolation, contributing to the onset of phenotypic modulation. Lastly, we acknowledge the impossibility to confirm the silencing of OMD at the protein level in SMC lysates, which may be due to the fact that OMD protein is rapidly secreted in the ECM after its synthesis in cells.

### 4.2 Conclusions

In summary, our translational approach integrating data from three patient cohorts with late‐stage CKD, CAVD and atherosclerosis, murine models of intimal and medial calcification and in vitro mechanistic studies, identified OMD as a novel factor broadly upregulated in both plasma and local tissue in association with cardiovascular calcification. We postulate that OMD is an important early modulator of cardiovascular calcification processes, enriched in association with vessel wall inflammation and osteoblastic transition of SMCs, with the capacity to attenuate ECM calcification. Mechanistically, the role of OMD is exerted likely through its link with TGFB1 signalling and SMAD3 transcription factor, and interplay with BMP2 in vascular tissues. Further studies are needed to elucidate the precise role of OMD in cellular transdifferentiation and ECM mineralisation.

### 4.3 Translational perspective

We conducted the first comprehensive, integrative analysis to elucidate the involvement and role of OMD in cardiovascular and CKD, specifically in association with calcification. Longitudinal studies in large cohorts that permit adjustment for traditional risk factors are needed to evaluate the potential of OMD as an early plasma biomarker of cardiovascular calcification and in association with clinical outcomes in patients. Our findings imply that the therapeutic potential of OMD as a target for inhibiting cardiovascular calcification should be investigated.

## CONFLICT OF INTERESTS

Grzegorz B. Wasilewski is employed by Nattopharma ASA, a company with interest in vitamin K. Peter Stenvinkel is on scientific advisory boards of Baxter, Vifor, REATA and Astra Zeneca. The funding bodies and companies had no involvement in the study design, manuscript writing or any other involvement in the creation of this manuscript.

## Supporting information

Supporting Information

## Data Availability

Material and Data pertaining to this manuscript are available from the corresponding author pending reasonable request. Restrictions associated with human biobank protection and personal data GDPR legislation will be respected.
