# Supplementary material for: Osteomodulin attenuates smooth muscle cell osteogenic transition in vascular calcification
Source: Clin Transl Med. 2022 Feb 20;12(2):e682. doi: 10.1002/ctm2.682 (PMC8858609; doi:10.1002/ctm2.682)
Supplement: Supplementary file 1 — Supporting Information [file CTM2-12-e682-s001.pdf]

Supplementary Material

Osteomodulin attenuates smooth muscle cell osteogenic transition in vascular calcification

Nikolaos-Taxiarchis Skenteris[1,2,3] Till Seime[2], Anna Witasp[4], Eva Karlöf[2], Grzegorz B. Wasilewski[3,5], Marina A. Heuschkel[3,6], Armand M.G. Jaminon[3], Loureen Oduor[7], Robert Dzhanaev[3,8], Malin Kronqvist[2], Mariette Lengquist[2], Frederique E.C.M. Peeters[9], Magnus Söderberg[10], Rebecka Hultgren[2], Joy Roy[2], Lars Maegdefessel[1,11], Hildur Arnardottir[1], Eva Bengtsson[7], Isabel Goncalves[7], Thomas Quertermous[12], Claudia Goettsch[6], Peter Stenvinkel[4], Leon J. Schurgers[3,13], Ljubica Matic[2#]

[1]Cardiovascular Medicine Unit, Department of Medicine, Karolinska Institute, Sweden; [2]Vascular Surgery, Department of Molecular Medicine and Surgery, Karolinska Institutet, Sweden; [3]Department of Biochemistry and CARIM, School for Cardiovascular Diseases, Maastricht University, Netherlands; [4]Division of Renal Medicine, Department of Clinical Sciences, Intervention and Technology, Karolinska Institutet, Sweden; [5]Nattopharma ASA, Oslo, Norway; [6]Department of Internal Medicine I-Cardiology, Medical Faculty, RWTH Aachen University; [7]Department of Clinical Sciences Malmö and Cardiology, Skåne University Hospital, Lund University, Sweden; [8]Helmholtz Institute for Biomedical Engineering, Biointerface Group, RWTH Aachen University, Aachen, Germany; [9]Department of Cardiology and CARIM, Maastricht University Medical Center+, School for Cardiovascular Diseases, Maastricht, Netherlands; [10]Cardiovascular, Renal and Metabolism Safety, Clinical Pharmacology and Safety Sciences, R&D, AstraZeneca, Gothenburg, Sweden; [11]Technical University Munich, Klinikum rechts der Isar, Department for Vascular and Endovascular Surgery, Germany; [12]Department of Cardiovascular Medicine, Stanford, USA; [13]Institute of Experimental Medicine and Systems Biology, RWTH Aachen University, Aachen, Germany

Supplementary Materials and Methods

Computed tomography (CT) angiography image analysis

Carotid plaques in BiKE cohort were assessed in pre-operative CT angiographies using a novel semi-automated, histopathologically validated software as previously described (vascuCAP, Elucid Bioimaging, Boston, MA)[1], rendering tissue and structural characteristics of the plaque, e.g., calcification volume proportion (CALCVolProp), lipid rich necrotic core volume proportion (LRNCVolProp), plaque-burden volume ratio and wall to lumen volume ratio.

Cardiac CT scans in CKD cohort were performed using a 64-channel detector scanner (Lightspeed VCT; General Electric (GE) Healthcare, Milwaukee, WI). CAC scores were expressed in Agatston units[2], the protocol and measurements as described previously in detail[2]. Total CAC score was calculated as the sum of CAC scores in the left main artery, the left anterior descending artery, the left circumflex artery, and the right coronary artery.

Animal material and studies

In general, the simple randomization method was applied in all animal studies and group results were analyzed in a blinded fashion. Male animals were used to ensure better control over the possible variability of data related to sex. There was no exclusion of animals from the study and analyses were conducted for all available samples in each experiment. All animal care and experimental procedures were performed in accordance with the guidelines for use of experimental animals and were approved by the local animal experimentation ethics committee.

Mouse atherosclerotic calcification model

Male C57BL6/J *ApoE*$^{-/-}$ mice (n=66) were purchased from Maastricht University and used in this study. Animals were 10 weeks of age when entering the study and housed in standard cages with free access to water and food. Atherosclerosis was induced as previously described[3] using a standard vitamin K-deficient Western type diet (WTD; 0.25% cholesterol, 15% cocoa butter and 1% corn oil; AB diets (4021.40), Woerden, the Netherlands). The control group additionally received vitamin K1 (100 µg/g) while the warfarin group received warfarin (3.0 mg/g) + vitamin K1 (1.5 mg/g), to avoid warfarin effects on the liver and prevent bleeding while introducing vitamin K-deficiency in the vasculature. Mice were sacrificed after 7, 13 or 19 weeks to perform immunohistochemical analysis. All animal studies were conducted under a protocol approved by the Ethics Committee for animal experiments of Maastricht University (DEC number 2011-014). Mouse aortic arch and the innominate artery were used for further immunohistochemistry analysis.

Rat model of medial vascular calcification

Sprague-Dawley rats (Charles River, Ecully, France) (n=90) 10 weeks of age and 220-250 gram were maintained in a controlled laboratory setting with water and food *ad libitum* (Teklad Diets, Madison WI, USA) and 12-hour day/night cycle. ¾ nephrectomy was performed to induce kidney failure[4]. One week later the animals were put on diet containing calcium 0.76%, phosphate 0.45%, 3 mg/g warfarin (Sigma) and 1.5 mg/g vitamin K1 (Sigma) to prime the rats for calcification and deplete the levels of vitamin K. Vitamin K1 was co-administrated with warfarin to avoid bleeding, yet having no effect in extra-hepatic tissues[5]. After three weeks the diet was changed to a purified diet consisting of calcium 1.34%, phosphate 1.2% and equal number of rats were allocated into high (100 µg/gram) and low (5 µg/gram) vitamin K2 (Nattopharma ASA, Oslo, Norway) groups for another 8 weeks. Animals were sacrificed and organs collected by flushing with isotonic buffer (40 mM Hepes, 150 mM NaCl, 5 mM KCl, 1 mM $MgCl_2$ and 2.5 mM $CaCl_2$, pH 7.3). Thoracic aortas were fixed over-night in 1% PFA and stored in 70% ethanol for embedding and analysis. Animal study protocol was approved by the Animal Experiments Committee of the VU University of Amsterdam and Maastricht Animal Care Committee (DEC2016-020).

***In vitro* experiments**

Human aortic smooth muscle cells (HAoSMCs) culture

Primary human aortic smooth muscle cells (HAoSMCs) were obtained from Lonza (#CC-2571, ascending aorta), maintained in Smooth muscle cell Growth Medium-2 BulletKit™ (SmGM, #CC-3182, Lonza) and used between passage 8-10. In addition, HAoSMCs were isolated from a non-diseased region of aortic tissue obtained from patients undergoing thoracic aortic aneurysm surgical repair in agreement with the Dutch Code for Proper Secondary Use of Human Tissue at Maastricht University and used between passage 5-8 for calcification assays. Briefly, intima, fat and connective tissue were carefully removed before cutting the sample into small fragments and placing into laminin (#L2020, Sigma) coated plates. The pieces were cultured in M199 medium (#22340-020, Gibco) containing 20% FBS, 1% PS and 1% amphotericin B (#15290-026, Gibco). When outgrowing cells reached confluency, they were passaged to laminin coated T25 flasks, tested for mycoplasma as well as immunohistochemically characterized for expression of classical SMC markers (CNN1, SM22a, α-SMA, p-MLC)[6].

HAoSMCs cytokine stimulation assays

HAoSMCs between passage 8-10 were plated on 6-well plates and left to adhere over-night in SmGM. The next day, the cells were treated with human cytokines: 20 ng/ml TGFβ1 (#T7039, Sigma), 150 ng/ml BMP2 (355-BEC-025/CF, R&D Biosystems), 20 ng/ml IL-4 (#204-IL-010, R&D Biosystems), 20 ng/ml IL-6 (#7270-IL-010/CF, R&D Biosystems) or 20 ng/ml IFNγ (#285-IF-100, R&D

Biosystems) in Opti-MEM™ Reduced Serum Medium, GlutaMAX™ supplement (#51985-026, Gibco) and collected at several time-points (12h, 24h, 48h and 72h) for RNA isolation and qPCR analyses. Full length rhOMD was purchased from R&D Systems (#2884-AD-050) and added to Opti-MEM culture medium at a concentration of 50 ng/ml (stock solution in PBS) for 24h. In addition, HAoSMCs between passage 8-10 were plated on 6-well plates and left to adhere over-night in SmGM. The next day, the cells were treated with human cytokines: 150 ng/ml BMP2 and 50 ng/ml rhOMD separate or in combination for 6 days.

Migration assay

To assess HAoSMC migration, an in vitro scratch assay was conducted as previously described[7]. Briefly, HAoSMCs passage 8-10 growing in 24-well plates were synchronized in Opti-MEM overnight. Thereafter, a straight scratch was created in a monolayer using a 1000 µL pipette tip and cells were washed once with PBS. Immediately, 50 ng/ml rhOMD was added for 48 hours in 5% FBS SmGM. Migration was continuously monitored, and images were acquired after 0, 2, 4, 8, 24, 32 and 48 hours. Wound closure was quantified by measuring the distance between the migration fronts at 3 random locations of 3 wells per time point and condition. Images were processed with Fiji Image J software.

Proliferation assay

HAoSMCs passage 8-10 were plated in 96-well plates (7500 cells per well) and left to adhere overnight in SmGM. After 6 h of serum starvation with Opti-MEM, cells were incubated in basal growth medium (5% FBS) with or without addition of 50 ng/ml rhOMD. Cell proliferation was assessed in a microplate reader *via* a colorimetric immunoassay based on BrdU incorporation during DNA synthesis (#11647229001, Roche, Basel, Switzerland) after 8, 16 and 24 hours, according to the manufacturer's protocol.

SMC calcification assays

HAoSMCs between passage 5-8 were seeded in 6-well plates and incubated in DMEM GlutaMAX (#31966-021; #12077549 Thermo Fisher) supplemented with 2.6 mM PO_4^{3-} (Pi)[8-11] and 10% FBS, for up to 12 days. Medium was refreshed every 3 days. Respective controls contained 2.5% or 10% FBS and MilliQ water. Cells were harvested after 3, 6, 9 and 12 days for RNA extraction. In some experiments, HAoSMCs between passage 8-10 were seeded on 6-well plates and left to adhere over-night in SmGM. The next day, cells were incubated in DMEM GlutaMAX supplemented with 2.6 mM Pi or 2.6 mM Pi and 50 ng/ml rhOMD or 150 ng/ml BMP2 or their combination in 5% FBS, for up to 12 days. Medium was refreshed every 3 days. Cells were harvested after 6 and 12 days for RNA extraction.

For calcification quantification, HAoSMCs between passage 5-8 were seeded with a density of 10.000 cells per cm^2 in 48-well plates and incubated in Medium 199 supplemented with 1% penicillin-streptomycin and 20% FBS for 24 hours. After 24 hours, medium was changed to control (growth medium) or high Pi (growth medium + 2.6 mM Pi) medium supplemented with 2.5% FBS. Quantification of deposited calcium was carried out using o-Cresolphthalein assay. Calcium measurements were normalized to protein content using microBCA protein assay kit.

Human primary coronary artery SMC culture and osteogenic transition

Human coronary artery smooth muscle cells (HCoSMCs, #C-12511, PromoCell) between passage 3-9 were grown in SMC growth medium 2 (SMC-GM2, #C-22062, PromoCell) supplemented with 0.5 ng/ml epidermal growth factor, 5 µg/ml insulin, 2 ng/ml basic fibroblast growth factor-β, and 5% FBS at 37 °C in humidified 5% CO_2. HCoSMCs were cultured for up to 14 days in the presence of either control medium (growth medium) or osteogenic medium (growth medium supplemented with 0.1 mM l-ascorbate phosphate, 10 mM β-glycerophosphate and 10 nM dexamethasone). Medium was refreshed every 3 days.

RNA interference

HAoSMCs were plated in 6 well plates at sub confluence (200.000 cells per well) and left to adhere over-night SmGM. Upon induction of osteogenic transformation by 20 ng/ml TGFb1 stimulation for 24 hours in OptiMEM medium, gene silencing was achieved via treatment with 50nM siRNA (SMAD3 #s8401[12] or scramble control #4390844, ThermoFisher) per well. SiRNA transfection was conducted by mixing with Lipofectamin (#15338100, ThermoFisher) and OptiMEM, allowing droplets to form for 30 minutes at room temperature. After 48 hours cells were harvested for RNA extraction. RNA silencing of HCoSMCs was performed as described previously[13]. Briefly, HCoSMCs were transfected using 20 nM siRNA against OMD (siOMD, #L-019921-01-0005, ON-TARGETplus Human OMD siRNA – SMARTpool, Horizon Discovery LTD, Cambridge, UK) or non-targeting siRNA (siSrc, #D-001810-01, ON-TARGETplus Non-targeting pool, Horizon Discovery LTD, Cambridge, UK) and DharmaFECT 1 (#T-2001-03, Horizon Discovery LTD, Cambridge, UK). Transfection was performed twice per week over the entire cell culture period in control and osteogenic medium.

Treatment with recombinant human Osteomodulin (rhOMD)

To evaluate the effect of OMD on calcification process, HAoSMCs between passage 5-8 were seeded in 48-well plates and incubated in Medium 199 supplemented with 1% penicillin-streptomycin and 2.5% FBS or 2.6 mM Pi for 12 days. Full length rhOMD was added to the culture medium at a concentration of 50 ng/ml (stock solution in PBS) simultaneously with 2.6 mM Pi for 12 days. The treatments were refreshed every 2-3 days. Calcification was assessed with the aforementioned protocol while for visualization, calcification nodules were assessed by a probe containing fetuin-A coupled with

Alexa Fluor 546[14, 15]. Sequential imaging was performed on a Cytation 3 System (BioSPX, Abcoude, The Netherlands).

Ex vivo human carotid atherosclerotic plaque culture

Human carotid atherosclerotic plaques were collected directly after carotid endarterectomy surgery at Karolinska Hospital. Each plaque was cut into small pieces (~2 mm^3), distributed into a Petri-dish, and thereafter incubated in RPMI 1640 medium (#31870-025, Gibco) supplemented with 1% penicillin-streptomycin and 10% FBS at 37°C in 5% CO_2 for 24h. The supernatant was collected and stored at -80°C until further assessment.

Molecular biology methods

Cytokine measurements

Supernatants from calcified HAoSMC were collected after 6, 9, 12 days of calcification assay in the different defined conditions, diluted 1:5 and analyzed for IL-1β concentration by ELISA (#DY201, R&D Systems) performed according to the manufacturer's instructions.

O-Cresolphthalein Assay

Cells in 48 well plates were washed twice with PBS. The mineralized matrix was dissolved in 0.1 M HCl for 15min. Randox O-Cresolphthalein kit (#CA 590, Randox, London, United Kingdom) was used according to the manufacturer's instruction to assess the amount of calcium embedded in extracellular matrix per sample. Values were converted to µg calcium and normalized with protein levels. All samples were assayed in triplicate.

MicroBCA Protein Assay

For normalization of the calcium content of the cells, microBCA protein assay kit (#23235, Thermo Fischer, Bleiswijk, the Netherlands) was performed. 0.1 M HCl cell suspension was neutralized and lysed with 0.1 M NaOH 0.2% SDS and incubated on a shaker for 20 mins. Plates were read at 750 nm using Cytation 3 (BioSPX, Abcoude, The Netherlands). Standard curve was created and sample absorbances were calculated, giving the protein content µg/µl protein. All samples were assayed in triplicate.

RNA extraction

RNA from BiKE tissues and HAoSMCs was prepared using Qiazol Lysis Reagent (Qiagen, Hilden, Germany) and purified by the RNeasy Mini kit (#74106, Qiagen), including DNase digestion. Total RNA from HCoSMCs was isolated using TriZol (Life Technologies). The concentration was measured using Nanodrop ND-2000 (Thermo Scientific, Waltham, MA) and quality estimated by a Bioanalyzer capillary electrophoresis system (Agilent Technologies, Santa Clara, CA).

Gene expression analyses by quantitative PCR (qPCR)

For qPCR, total RNA was reverse-transcribed using High-Capacity RNA-to-cDNA kit (#4387406, Applied Biosystems, Carlsbad, CA). PCR amplification was performed in 384-well plates in 7900 HT real-time PCR system (Applied Biosystems), using TaqMan® Universal PCR Master Mix (#4324018, Applied Biosystems) and TaqMan® Gene Expression Assays (ACTA2 probe Hs00426835_g1, BMP2 probe HS00154192_m1, CNN1 probe Hs00154543_m1, CASP3 probe Hs00234387_m1, CD68 probe Hs02836816_g1, CDH2 probe Hs00983056_m1, COL4A4 probe Hs01011868_m1, COL10A1 probe Hs00166657_m1, FN1 probe Hs01549976_m1, HPSE probe Hs00935036_m1, MYH11 probe Hs00975796_m1, MYOCD probe Hs00538076_m1, OMD probe Hs01060466_m1, Hs00192325_m1, RUNX2 probe Hs01047973_m1, SMAD3 probe Hs00969210_m1, SOX9 probe Hs00165814_m1, TNFAIP6 probe Hs00200180_m1, purchased from Thermo Fisher). All samples were measured in duplicates. Results were normalized to the equal mass of total RNA as well as the Ct values of RPLPO (Hs99999902_m1) housekeeping control. The relative amount of target gene mRNA was calculated by the 2^(-ΔΔCt) method.

Gene expression analyses by microarrays

Gene expression analyses of n=127 endarterectomy samples were performed in two batches using Affymetrix HG-U133 plus 2.0 Genechip arrays (Santa Clara, CA, USA). Robust multi-array average normalization, filtering of probe sets based on signal intensity and batch effect correction were performed, and processed gene expression data were recorded on a log2 scale as previously described[16]. The full data set is available from Gene Expression Omnibus (accession number GSE21545). The BiKE study cohort demographics **(Table S3)**, details of sample processing, and full microarray analyses have been previously described[16, 17].

For gene expression analysis of plaques in the calcification context, RNA was extracted from n=40 BiKE carotid plaques where calcification grade was estimated by TeraRecon software after processing the CT images, and microarrays were performed using Affymetrix HTA 2.0 arrays (Affymetrix, Santa Clara, CA). Only RNA material of suitable quality with respect to purity and integrity (RIN 7-10, $A_{260\text{-}280}$1.7-2.0, $A_{260/230}$0.7-1.5) was used and all samples were analyzed in 1 batch. Annotation was based on the Hg19 genome build, NCBI genome version GRCh37 and NetAffx build 34, for all 70523 probe sets. Robust multiarray average (RMA) normalization was performed and processed gene expression data was returned in log2-scale as previously described[16]. This microarray dataset is available from Gene Expression Omnibus (accession number GSE125771). This BiKE's study sub-cohort demographics, details of sample processing, and full microarray analyses have also been previously described[18].

For gene expression analysis of HCoSMCs and HAoSMCs in osteogenic medium, RNA was extracted from n=3 independent experiments and microarrays were performed using Affymetrix HTA 2.0 arrays

(Affymetrix, Santa Clara, CA). Only RNA material of suitable quality with respect to purity and integrity (RIN 9-10, $A_{260\text{-}280}$ 1.7-2.0, $A_{260/230}$ 0.7-1.5) was used and all samples were analyzed in 1 batch. Discovery analyses of significantly dysregulated genes were performed by comparing the differential expressed genes (DEGs) in HCoSMCs treated with siRNA for OMD (n=3) *vs.* scramble control (n=3) in osteogenic medium for 14 days, or alternatively in HAoSMCs treated with rhOMD (n=3) *vs.* control (n=3) in osteogenic medium for 6 days. Following DEG analysis, gene set enrichment analyses on Reactome terms were performed using public ENRICHR software (http://amp.pharm.mssm.edu/Enrichr). In all analyses adjusted $p < 0.05$ was considered to indicate statistical significance.

Single cell RNA sequencing data analysis of human coronary and carotid artery plaques

Data used in this study was published previously[19, 20] processed and plotted using PlaqView public software (https://millerlab.shinyapps.io/PlaqView/)[21]. Briefly, coronary arteries were dissected from explanted hearts of transplant recipients who provided their written consent prior to the procedure. Tissues were obtained from the Human Biorepository Tissue Research Bank under the Department of Cardiothoracic Surgery, Stanford with approval from the Stanford University Institutional Review Board. The basic clinical characteristics of the patients included (n=4) in the study were previously described[19]. The top 100 differently expressed gene markers were used to distinguish each cell cluster in the human scRNAseq dataset.

Histochemistry

In general, prior to all stainings, tissue specimens were deparaffinized in 2 changes of xylene (VWR, USA) for 2 minutes. Thereafter tissue specimens were re-hydrated in 2 consecutive changes of ethanol series (100%, 96%, 70% and 50% ethanol, 2 minutes each) and briefly washed in distilled water.

To enable further processing for histology, macro-calcified plaques were de-calcified after fixation in Modified Decalcification Solution (Histolab, Gothenburg, Sweden, #HL24150.1000) for 4-6 days depending on plaque size. Plaques were then rinsed in distilled water, dehydrated and paraffin-embedded.

Alizarin Red and von Kossa stainings

Adjacent sections were stained using histochemistry techniques for assessing calcification (von Kossa; VK and Alizarin Red; AR). For VK in brief, tissue sections were rehydrated and incubated in 1% $AgNO_3$. Next, for 1 minute in sodaformol followed by 5 minutes of 5% $Na_2S_2O_3*H_2O$. Counterstain was performed in Nuclear Fast Red, sections were dehydrated and covered in entallan. Alizarin Red S (Sigma-Aldrich) staining was performed according to the standard staining protocol for calcium deposits. The slides were deparaffinized with Histolab Clear and rehydrated with ethanol. Slides were

washed and stained with Alizarin Red solution (Sigma-Aldrich), which stains calcium in red-orange. Slides were dehydrated in acetone (Sigma-Aldrich) then in acetone-xylene (1:1), thereafter rehydrated and mounted.

For quantification of the cell culture AR staining, 100 mM cetylpyridinium chloride (Sigma-Aldrich) in 10 mM sodium phosphate buffer (Roth) was added to each well and incubated for 20 min at 37°C. The eluted stain was measured at 570 nm using a spectrophotometer.

Antibodies

The following primary antibodies were used in the study: anti-OMD (ab154249, Abcam and AF3308, R&D Systems), antibodies for cell-specific markers were smooth muscle α-actin (α-SMA, ab15734, Abcam) and RUNX2 (M70, sc10758, Santa Cruz Biotechnology and AMAB90591, Sigma).

Immunohistochemistry (IHC)

For staining of human tissues, IHC reagents were from Biocare Medical. Isotype rabbit and mouse IgG were used as negative controls. In brief, 5 μm sections were deparaffinized in Histolab Clear and rehydrated in ethanol. For antigen retrieval, slides were subjected to high-pressure boiling in DIVA buffer (pH 6.0) or TE buffer (pH 9.0). After blocking with Background Sniper, primary antibodies diluted in Da Vinci Green solution were applied and incubated at room temperature for 1 hour. A double-stain probe-polymer detection kit (Mach 2) containing both alkaline phosphatase and horseradish peroxidase was applied, with subsequent detection using Warp Red and Vina Green. All slides were counterstained with Hematoxylin QS (Vector laboratories, Burlingames, USA), dehydrated and mounted in Pertex (Histolab, Gothenburg, Sweden). Images were taken using a Nikon OPTIPHOT-2 microscope equipped with digital camera and NIS-Elements software.

For staining of mouse, rat and human aortic valve sections, sequential 5 μm slides were rehydrated, and antigens were retrieved by boiling in TriSodiumCitrate buffer (pH 6.0). Primary antibodies were applied and incubated over-night at 4°C and then visualized with a Nova-RED substrate (Vector #SK-4800, Vector Laboratories, Inc). Sections were counterstained with hematoxylin (Klinipath, #4085-9002) and mounted with entellan (Merck #7961). In negative controls, generic IgG primary antibody was used. Prior to tissue analyses, whole tissue reference images were taken with the VENTANA iScan HT slide scanner (Ventana medical systems Inc, Roche). Image J v2.0 software was employed to quantify mean intensity of α-SMA, OMD, RUNX2 staining. At least 3 fields of view were quantified and averaged per staining per animal of 3 rats and 3-4 mice were used per condition.

Supplementary References:

1. Sheahan M, Ma X, Paik D, Obuchowski NA, St Pierre S, Newman WP, 3rd, Rae G, Perlman ES, Rosol M, Keith JC, Jr. and Buckler AJ. Atherosclerotic Plaque Tissue: Noninvasive Quantitative Assessment of Characteristics with Software-aided Measurements from Conventional CT Angiography. *Radiology*. 2018;286:622-631.
2. Agatston AS, Janowitz WR, Hildner FJ, Zusmer NR, Viamonte M, Jr. and Detrano R. Quantification of coronary artery calcium using ultrafast computed tomography. *Journal of the American College of Cardiology*. 1990;15:827-32.
3. Schurgers LJ, Joosen IA, Laufer EM, Chatrou MLL, Herfs M, Winkens MHM, Westenfeld R, Veulemans V, Krueger T, Shanahan CM, Jahnen-Dechent W, Biessen E, Narula J, Vermeer C, Hofstra L and Reutelingsperger CP. Vitamin K-Antagonists Accelerate Atherosclerotic Calcification and Induce a Vulnerable Plaque Phenotype. *PLOS ONE*. 2012;7:e43229.
4. Neradova A, Wasilewski G, Prisco S, Leenders P, Caron M, Welting T, van Rietbergen B, Kramann R, Floege J, Vervloet MG and Schurgers LJ. Combining phosphate binder therapy with vitamin K2 inhibits vascular calcification in an experimental animal model of kidney failure. *Nephrology, dialysis, transplantation : official publication of the European Dialysis and Transplant Association - European Renal Association*. 2021.
5. Shobeiri N, Pang J, Adams MA and Holden RM. Cardiovascular disease in an adenine-induced model of chronic kidney disease: the temporal link between vascular calcification and haemodynamic consequences. *Journal of Hypertension*. 2013;31.
6. Furmanik M, Chatrou M, van Gorp R, Akbulut A, Willems B, Schmidt H, van Eys G, Bochaton-Piallat M-L, Proudfoot D, Biessen E, Hedin U, Perisic L, Mees B, Shanahan C, Reutelingsperger C and Schurgers L. Reactive Oxygen-Forming Nox5 Links Vascular Smooth Muscle Cell Phenotypic Switching and Extracellular Vesicle-Mediated Vascular Calcification. *Circulation research*. 2020;127:911-927.
7. Liang C-C, Park AY and Guan J-L. In vitro scratch assay: a convenient and inexpensive method for analysis of cell migration in vitro. *Nature Protocols*. 2007;2:329-333.
8. Jono S, McKee MD, Murry CE, Shioi A, Nishizawa Y, Mori K, Morii H and Giachelli CM. Phosphate regulation of vascular smooth muscle cell calcification. *Circulation research*. 2000;87:E10-7.
9. Hortells L, Sosa C, Millán Á and Sorribas V. Critical Parameters of the In Vitro Method of Vascular Smooth Muscle Cell Calcification. *PLoS One*. 2015;10:e0141751.
10. Seime T, Akbulut AC, Liljeqvist ML, Siika A, Jin H, Winski G, van Gorp RH, Karlöf E, Lengquist M, Buckler AJ, Kronqvist M, Waring OJ, Lindeman JHN, Biessen EAL, Maegdefessel L, Razuvaev A, Schurgers LJ, Hedin U and Matic L. Proteoglycan 4 Modulates Osteogenic Smooth Muscle Cell Differentiation during Vascular Remodeling and Intimal Calcification. *Cells*. 2021;10.
11. Zhang D, Bi X, Liu Y, Huang Y, Xiong J, Xu X, Xiao T, Yu Y, Jiang W, Huang Y, Zhang J, Zhang B and Zhao J. High Phosphate-Induced Calcification of Vascular Smooth Muscle Cells is Associated with the TLR4/NF-κb Signaling Pathway. *Kidney and Blood Pressure Research*. 2017;42:1205-1215.
12. Iyer D, Zhao Q, Wirka R, Naravane A, Nguyen T, Liu B, Nagao M, Cheng P, Miller CL, Kim JB, Pjanic M and Quertermous T. Coronary artery disease genes SMAD3 and TCF21 promote opposing interactive genetic programs that regulate smooth muscle cell differentiation and disease risk. *PLOS Genetics*. 2018;14:e1007681.
13. Goettsch C, Hutcheson JD, Aikawa M, Iwata H, Pham T, Nykjaer A, Kjolby M, Rogers M, Michel T, Shibasaki M, Hagita S, Kramann R, Rader DJ, Libby P, Singh SA and Aikawa E. Sortilin mediates vascular calcification via its recruitment into extracellular vesicles. *The Journal of clinical investigation*. 2016;126:1323-1336.
14. Jahnen-Dechent W, Heiss A, Schäfer C and Ketteler M. Fetuin-A regulation of calcified matrix metabolism. *Circulation research*. 2011;108:1494-509.
15. Jaminon AMG, Akbulut AC, Rapp N, Kramann R, Biessen EAL, Temmerman L, Mees B, Brandenburg V, Dzhanaev R, Jahnen-Dechent W, Floege J, Uitto J, Reutelingsperger CP and Schurgers

LJ. Development of the BioHybrid Assay: Combining Primary Human Vascular Smooth Muscle Cells and Blood to Measure Vascular Calcification Propensity. *Cells*. 2021;10:2097.

16. Perisic L, Aldi S, Sun Y, Folkersen L, Razuvaev A, Roy J, Lengquist M, Åkesson S, Wheelock CE, Maegdefessel L, Gabrielsen A, Odeberg J, Hansson GK, Paulsson-Berne G and Hedin U. Gene expression signatures, pathways and networks in carotid atherosclerosis. *Journal of Internal Medicine*. 2016;279:293-308.

17. Perisic L, Hedin E, Razuvaev A, Lengquist M, Osterholm C, Folkersen L, Gillgren P, Paulsson-Berne G, Ponten F, Odeberg J and Hedin U. Profiling of Atherosclerotic Lesions by Gene and Tissue Microarrays Reveals PCSK6 as a Novel Protease in Unstable Carotid Atherosclerosis. *Arteriosclerosis, thrombosis, and vascular biology*. 2013;33:2432-2443.

18. Karlöf E, Seime T, Dias N, Lengquist M, Witasp A, Almqvist H, Kronqvist M, Gådin JR, Odeberg J, Maegdefessel L, Stenvinkel P, Matic LP and Hedin U. Correlation of computed tomography with carotid plaque transcriptomes associates calcification with lesion-stabilization. *Atherosclerosis*. 2019;288:175-185.

19. Wirka RC, Wagh D, Paik DT, Pjanic M, Nguyen T, Miller CL, Kundu R, Nagao M, Coller J, Koyano TK, Fong R, Woo YJ, Liu B, Montgomery SB, Wu JC, Zhu K, Chang R, Alamprese M, Tallquist MD, Kim JB and Quertermous T. Atheroprotective roles of smooth muscle cell phenotypic modulation and the TCF21 disease gene as revealed by single-cell analysis. *Nat Med*. 2019;25:1280-1289.

20. Pan H, Xue C, Auerbach BJ, Fan J, Bashore AC, Cui J, Yang DY, Trignano SB, Liu W, Shi J, Ihuegbu CO, Bush EC, Worley J, Vlahos L, Laise P, Solomon RA, Connolly ES, Califano A, Sims PA, Zhang H, Li M and Reilly MP. Single-Cell Genomics Reveals a Novel Cell State During Smooth Muscle Cell Phenotypic Switching and Potential Therapeutic Targets for Atherosclerosis in Mouse and Human. *Circulation*. 2020;142:2060-2075.

21. Ma WF, Hodonsky CJ, Turner AW, Wong D, Song Y, Barrientos NB and Miller CL. Single-cell RNA-seq analysis of human coronary arteries using an enhanced workflow reveals SMC transitions and candidate drug targets. *bioRxiv*. 2020:2020.10.27.357715.

Supplementary Figures

Supplementary Figure I

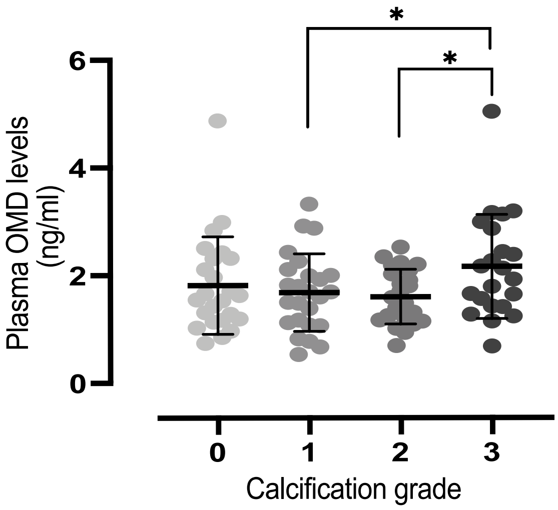

Figure S1. OMD protein measurements in plasma from CKD patients stratified in groups according to the medial calcification grade/score (CS) of epigastric arteries from these patients (ranging from 0-3, where 0 signifies no arterial calcification, 1 and 2 refer to mild and moderate calcification, while 3 refers to extensive arterial calcification). Number of patients per group: n=25 for CS=0, n=25 for CS=1, n=24 for CS=2, n=24 for CS=3. One-way ANOVA multiple comparison test; data presented as mean with SD. Differences between groups were considered significant at *P* values < 0.05 (**P* < 0.05).

Supplementary Figure II

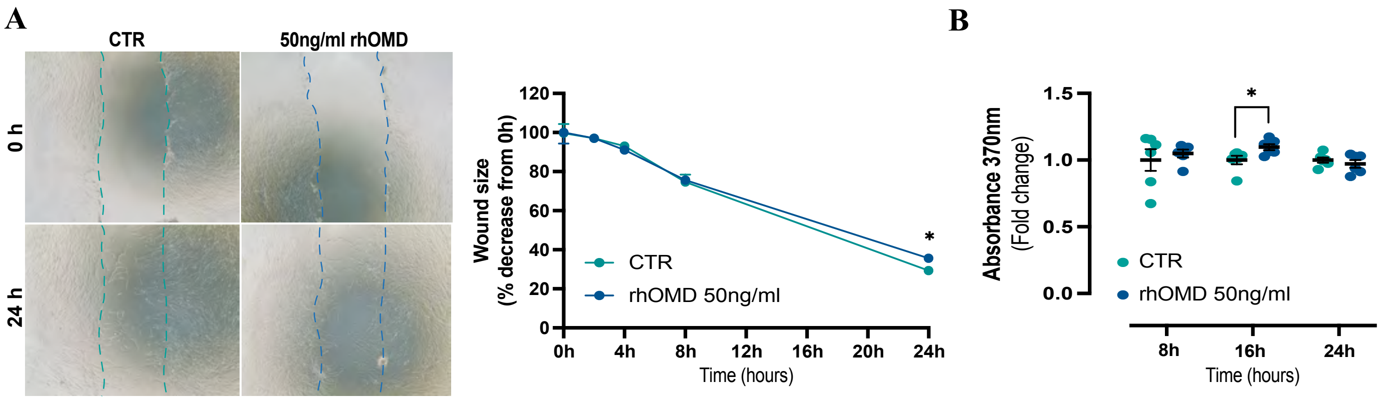

Figure S2. Effect of full length rhOMD on cell migration and proliferation. **A)** Representative images and quantification of a scratch wound healing assay on HAoSMCs treated with 50 ng/ml rhOMD for up to 48 hours. Normalization performed from images acquired immediately after scratch (0 h) representing 100% of the wound area. At least 2 optical fields quantified per well of at least n=3 wells per condition. **B)** BrdU proliferation assay of HAoSMCs treated with 50 ng/ml rhOMD for 24 hours. N=3 independent experiments in duplicates. Statistical significance between groups was assessed by Student's t-test; data expressed as mean with SEM. Differences between groups were considered significant at *P* values < 0.05 (**P* < 0.05).

Supplementary Figure III

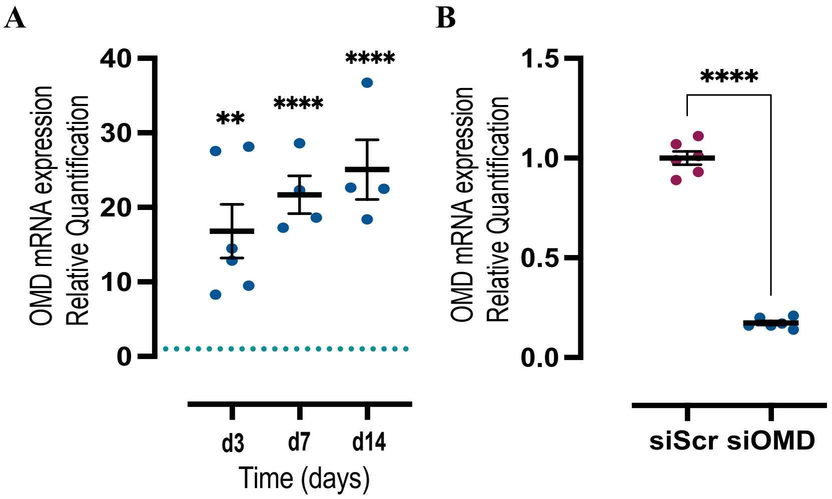

Figure S3. A) OMD mRNA expression levels in HCoSMCs treated with osteogenic medium consisting of 0.1 mM l-ascorbate phosphate, 10 mM β-glycerophosphate and 10 nM dexamethasone for 3, 7 and 14 days, for promoting the osteoblast phenotype. The values of OMD gene expression in osteogenic medium treated HCoSMCs were normalized to the corresponding values of non-treated cells (green dotted line). **B)** OMD mRNA expression levels in HCoSMCs treated with siRNA for OMD or scramble control in osteogenic medium for 14 days. N=3 independent experiments in duplicates in both A and B. Statistical significance between groups was assessed by Student's t-test; data expressed as mean with SEM. Differences between groups were considered significant at *P* values < 0.05 (***P* ≤ 0.01, *****P* ≤ 0.0001).

Supplementary Figure IV

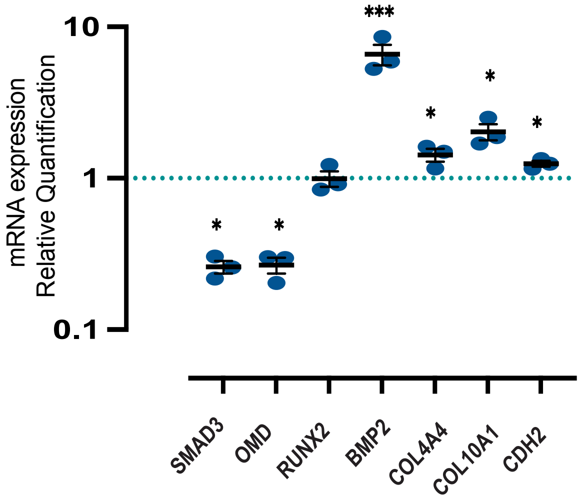

Figure S4. Gene expression analysis of SMAD3, OMD, RUNX2, BMP2 and extracellular matrix proteins COL4A4, COL10A1 and CDH2 in HAoSMCs stimulated with TGFβ1 for 24 hours followed by SMAD3 gene silencing for additional 48 hours. N=3 independent experiments and data expressed as mean with SEM. Statistical significance between groups was assessed by Student t-test comparing siSMAD3 *vs.* scramble control (Scr; green dotted line). Differences between groups were considered significant at *P* values < 0.05 (*$P < 0.05$, ***$P \leq 0.001$).

Supplementary Figure V

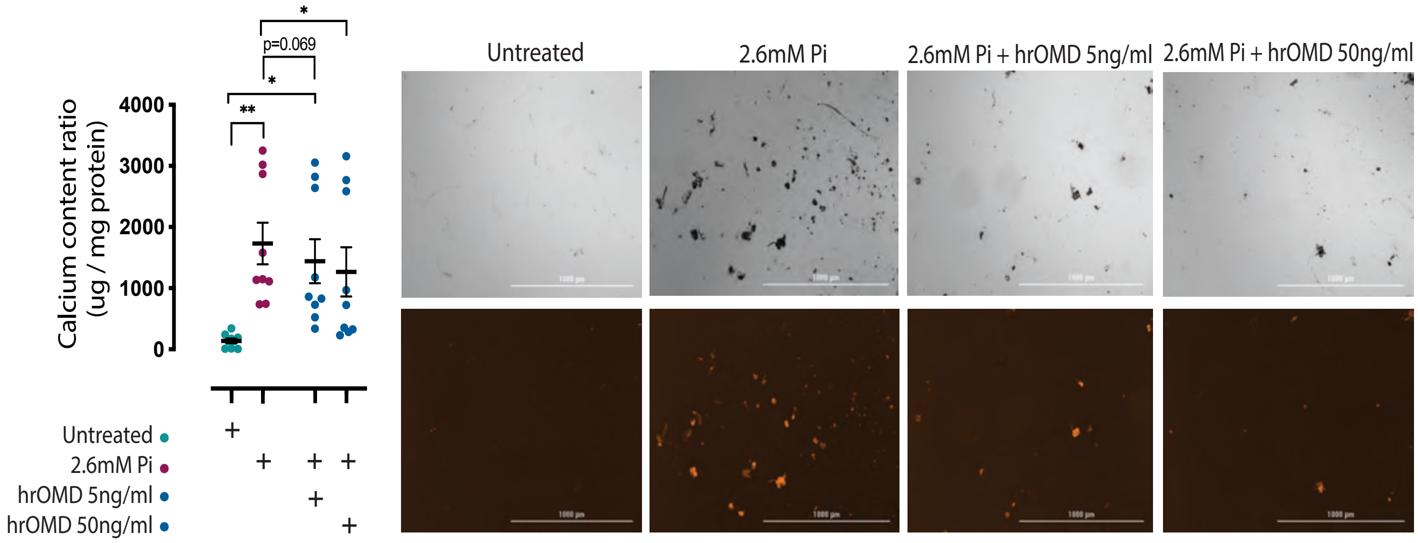

Figure S5. Quantification of the in vitro calcification of primary HAoSMCs treated with 2.6mM Pi for 12 days in the absence or presence of two concentrations of human OMD protein. The experiment was performed in triplicate with cells from 3 patients. Statistical difference between groups assessed by one-way ANOVA; data expressed as mean with SEM. Representative images of the calcification assay where calcification was visualized by a probe containing fetuin-A coupled with Alexa Fluor 546. Differences between groups were considered significant at *P* values < 0.05 (*P < 0.05, **P ≤ 0.01).

Supplementary Figure VI

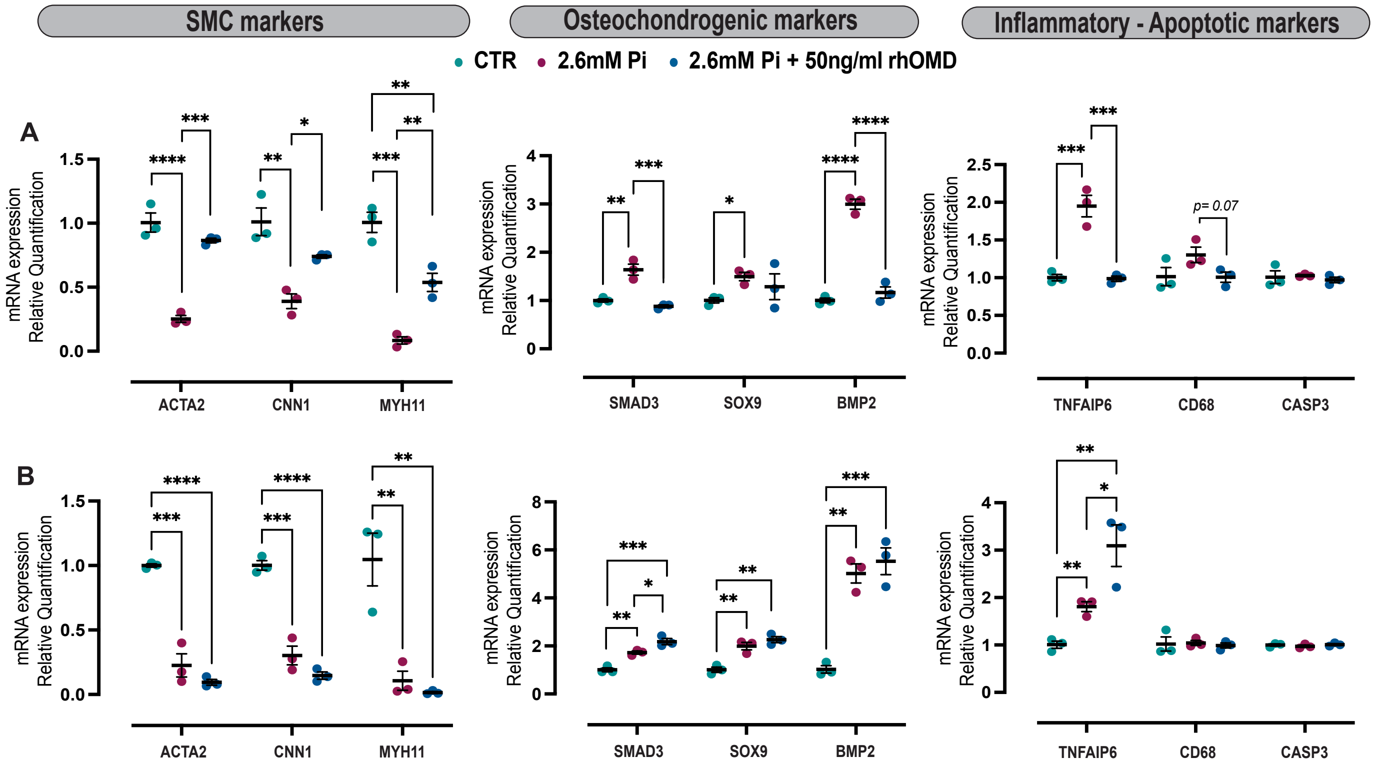

Figure S6. Gene expression analysis of typical SMC markers, osteochondrogenic and inflammatory markers in HAoSMCs treated with 2.6 mM Pi or 2.6 mM Pi + 50 ng/ml rhOMD for **(A)** 6 days and **(B)** 12 days. Statistical significance between groups was assessed by one-way ANOVA multiple comparison test; data expressed as mean with SEM. Differences between groups were considered significant at P values < 0.05 (*$P < 0.05$, **$P \leq 0.01$, ***$P \leq 0.001$, ****$P \leq 0.0001$).

Supplementary Figure VII

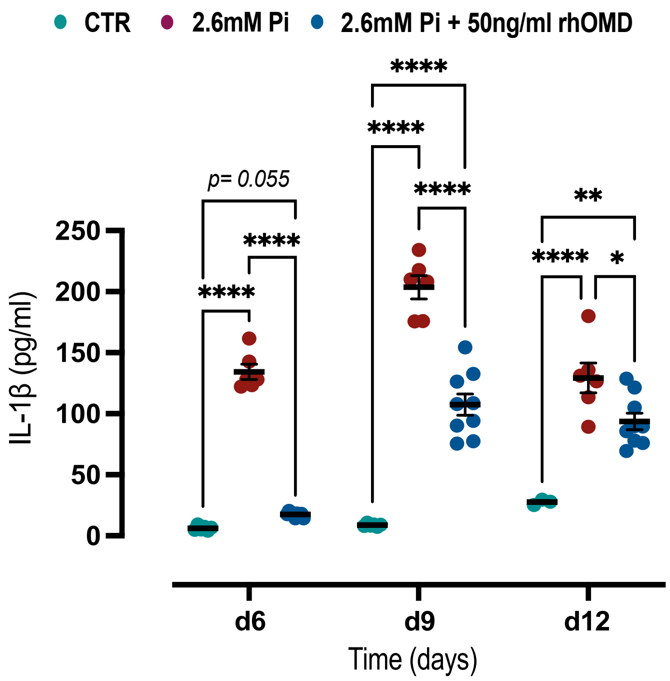

Figure S7. IL-1β protein secretion in the supernatants of HAoSMCs in control (untreated) and treated conditions with 2.6 mM Pi or 2.6 mM Pi + 50 ng/ml rhOMD after 6, 9 and 12 days. Statistical significance between groups was assessed by one-way ANOVA multiple comparison test; data expressed as mean with SEM. Differences between groups were considered significant at P values < 0.05 (*P < 0.05, **P ≤ 0.01, ****P ≤ 0.0001).

Supplementary Figure VIII

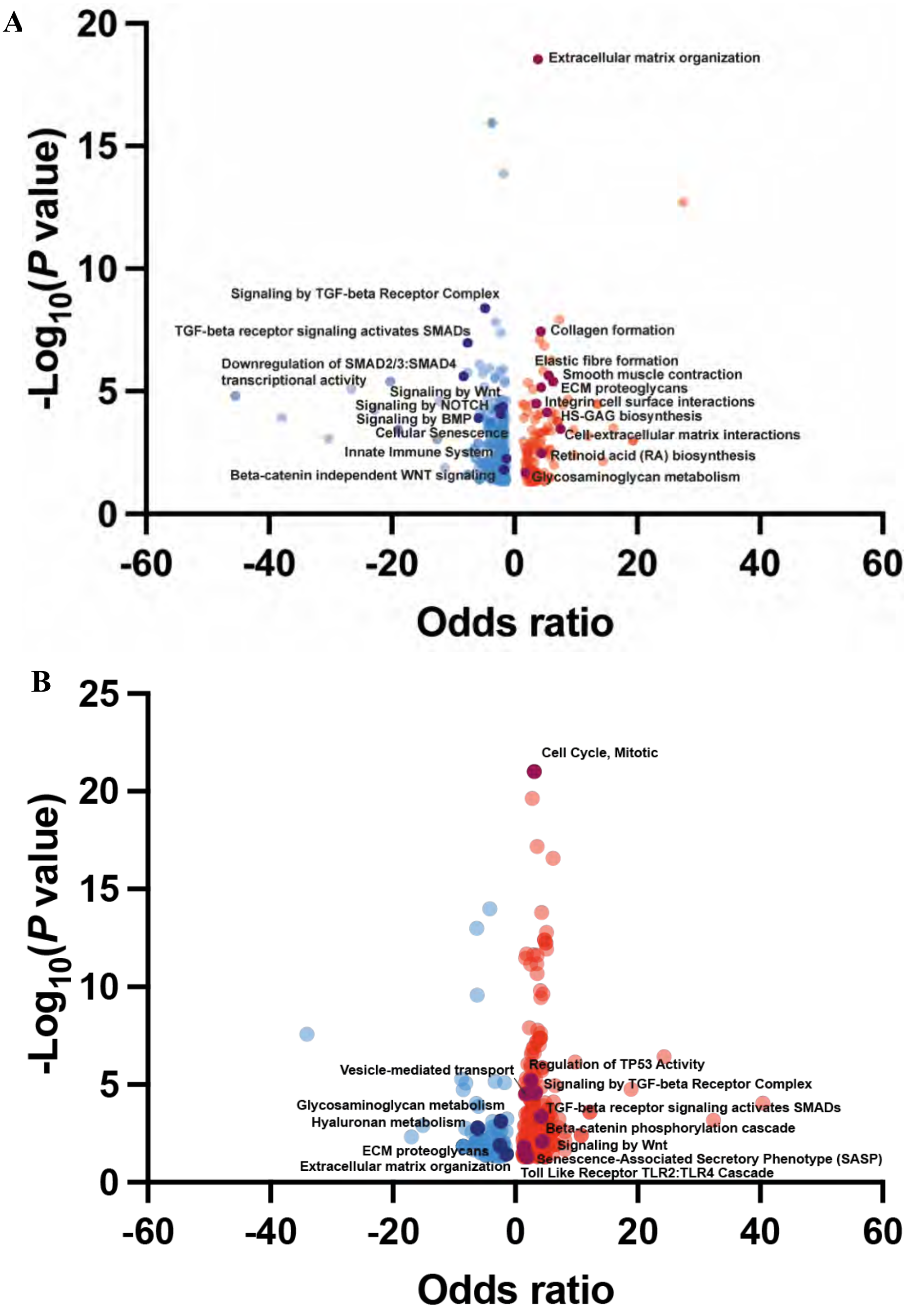

Figure S8. A) Volcano plot showing the top significantly downregulated (blue) and upregulated (red) Reactome pathways comparing HAoSMCs treated with rhOMD (n=3) *vs.* control (n=3) in osteogenic medium for 6 days. **B)** Volcano plot showing the top significantly downregulated (blue) and upregulated (red) Reactome pathways comparing HCoSMCs treated with siOMD (n=3) *vs.* control (siScr, n=3) in osteogenic medium for 14 days.

Supplementary Figure IX

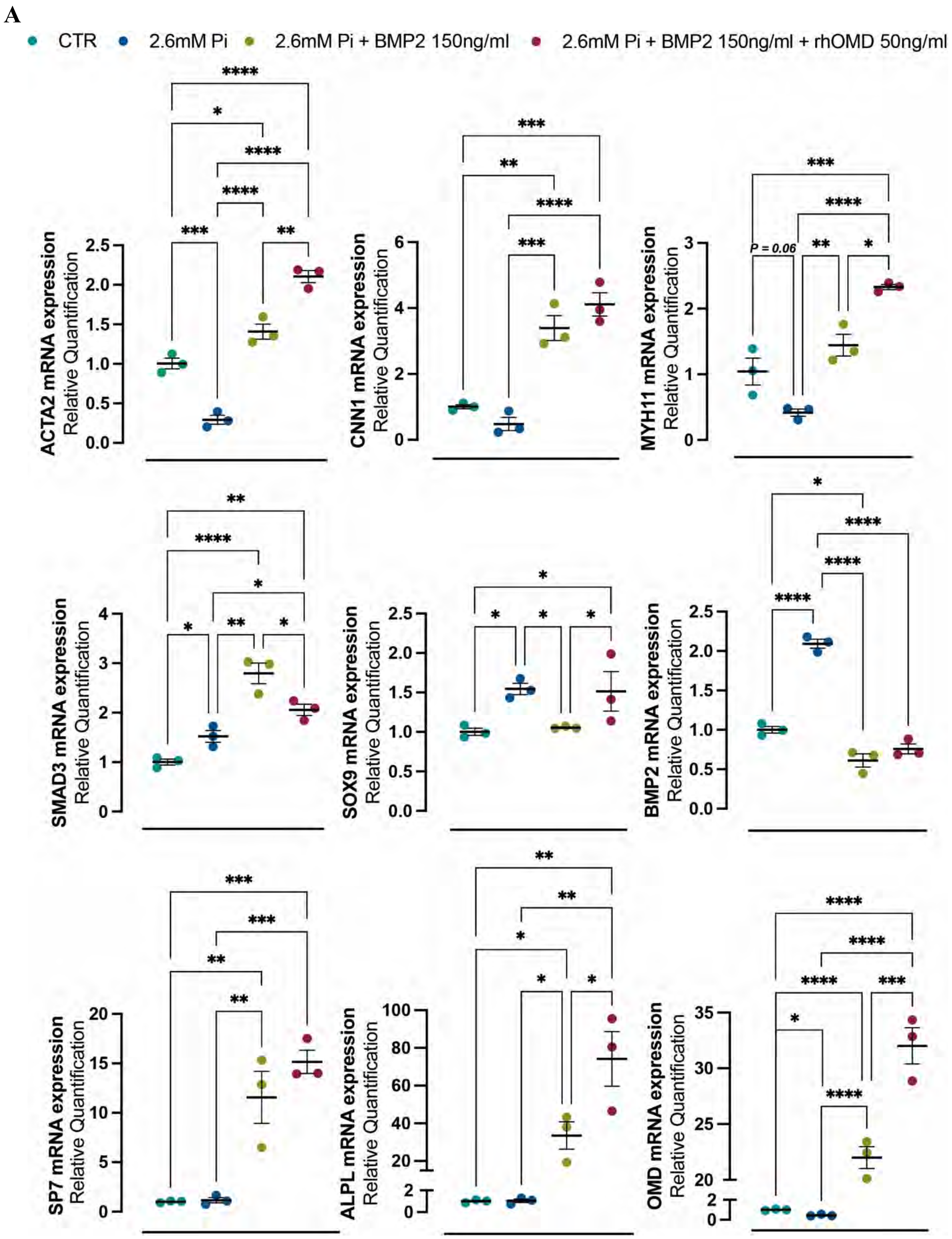

B

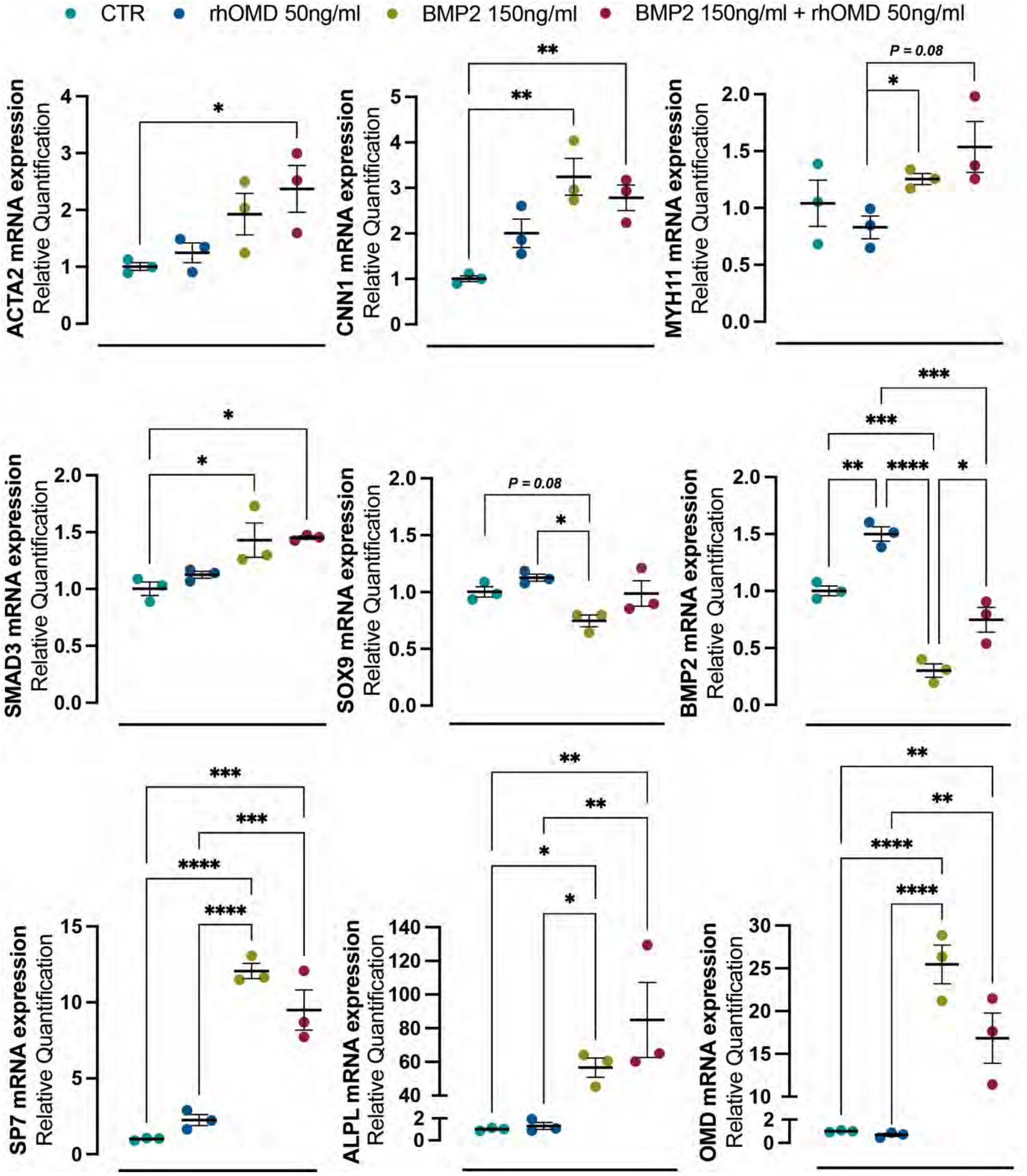

CTR
rhOMD 50ng/ml
BMP2 150ng/ml
BMP2 150ng/ml + rhOMD 50ng/ml
ACTA2 mRNA expression
CNN1 mRNA expression
MYH11 mRNA expression
SMAD3 mRNA expression
SOX9 mRNA expression
BMP2 mRNA expression
SP7 mRNA expression
ALPL mRNA expression
OMD mRNA expression
Relative Quantification
P = 0.08

469

C

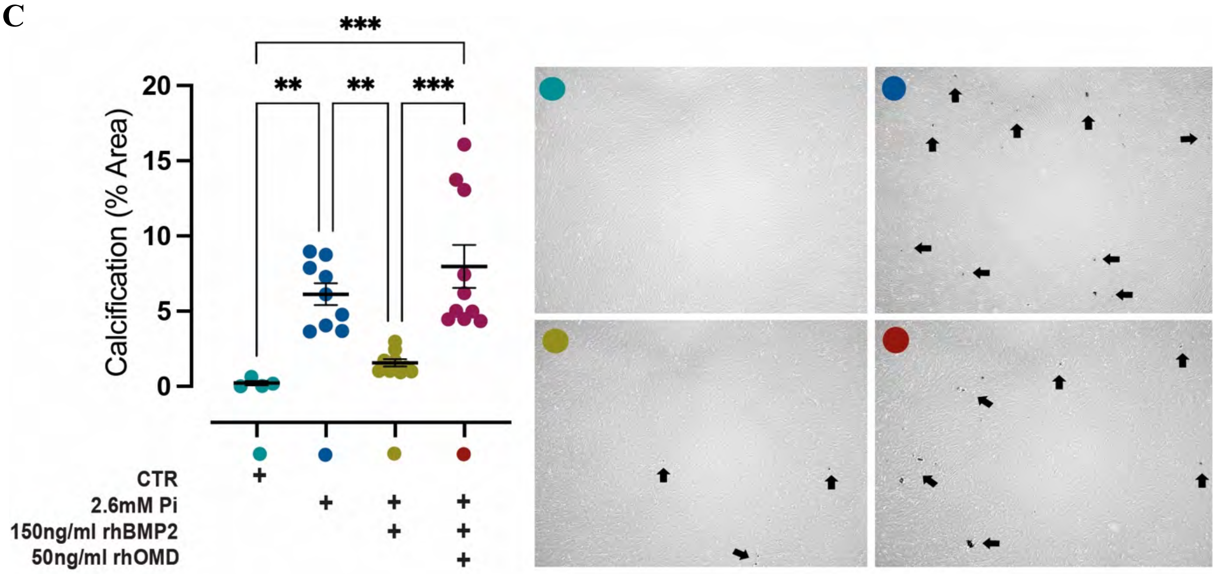

Figure S9. Gene expression analysis of typical SMC markers and osteochondrogenic markers in HAoSMCs treated with 50 ng/ml rhOMD or 150ng/ml BMP2 or their combination **(A)** and under high Pi osteogenic conditions **(B)** for 6 days. Statistical significance between groups was assessed by Student t-test and one-way ANOVA multiple comparison test; data expressed as mean with SEM. **C)** Quantification of the in vitro calcification of HAoSMCs treated with 2.6mM Pi for 6 days in combination with 150ng/ml BMP2 and 50ng/ml OMD proteins. Representative images of the calcification assay. Quantification performed with Image J and calcified nodules were quantified as percentage of calcification per optical field area. Statistical difference between groups assessed by one-way ANOVA; data expressed as mean with SEM. Differences between groups were considered significant at *P* values < 0.05 (**P* < 0.05, ***P* ≤ 0.01, ****P* ≤ 0.001, *****P* ≤ 0.0001).

Supplementary Tables

Supplementary Table I

	Calcification score				
	**0**	**1**	**2**	**3**	**P-value**
N	25	25	24	24	
Age, *years median (range)*	**31 (22-63)**	**51 (45-69)**	**56 (45-69)**	**55 (45-71)**	**<0.0001**
Sex *N males (%)*	14 (56)	18 (72)	19 (79)	20 (83)	0.1474
BMI, *kg/m² median (range)*	**22.6 (17.7-33.5)**	**25.6 (18.1-38.9)**	**25.4 (22.3-29.3)**	**25.0 (19.8-30.2)**	**0.0287**
Diabetes *N yes (%)*	**1 (5)**	**0 (0)**	**5 (22)**	**11 (46)**	**0.0002**
Smoking *N yes (%)*	0 (0)	0 (0)	1 (5)	0 (0)	0.3794
Cardiac CT, *CAC median (range)*	**0 (0-336)**	**49 (0-1312)**	**31 (0-4278)**	**973 (3775)**	**<0.0001**
Statins *N yes (%)*	**6 (24)**	**10 (40)**	**10 (42)**	**15 (63)**	**0.0572**

Table S1. Demographics of the end-stage renal disease patients from the CKD cohort used in plasma analysis. Calcification score based on the histological assessment of epigastric arteries.

Supplementary Table II

General	Whole cohort, n (%)	Asymptomatic	Symptomatic	P-value
N	85	28	57	
Age *(years, mean)*	**70.9**	**72.2**	**68.3**	**0.027**
Gender *(female/male)*	**23/63 (27/73)**	**2/26 (7.1/92.9)**	**21/36 (36.8/63.2)**	**0.0039**
BMI *(mean)*	26.59	26.52	26.72	ns
Smoking *(Yes/No)*	14/66 (12.5/80.5)	4/24 (14.3/85.7)	10/42 (19.3/77.3)	ns
**Comorbidities**				
Previous myocardial infarction	14 (17.3)	3 (11.1)	11 (20.4)	ns
Previous TIA/Stroke	**21 (25.2)**	**11 (39.3)**	**10 (18.2)**	**0.059**
Angina pectoris	17 (21.2)	6 (22.2)	11 (20.7)	ns
Diabetes	18 (21.6)	5 (22.2)	13 (22.8)	ns
Inflammatory diseases (rheumatism, psoriasis, multiple sclerosis, inflammatory bowel disease)	7 (8.7)	2 (7.4)	5 (9.3)	ns
**Therapy**				
Lipid decreasers (ezetimib, HMG-CoA reductase inhibitors)	80 (96.4)	27 (96.4)	53 (96.4)	ns
Antihypertensives (ACE inhibitors, beta-blockers, diuretics, angiotensine II blockers	66 (77.6)	22 (78.6)	44 (77.2)	ns
**Symptoms**				
Amaurosis fugax (AF)	*16 (18.8)*	*0 (0)*	*16 (28)*	
Transitory ischemic attack (TIA)	*23 (27)*	*0 (0)*	*23 (40.3)*	
Minor Stroke (MS)	*17 (20)*	*0 (0)*	*17 (29.8)*	
**Lab measurements (mean)**				
Serum creatinine (mg/dl)	**83.2**	**90.3**	**79.5**	**0.03**
Serum cholesterol (mmol/l)	4,3	4.0	4.5	ns
LDL (mmol/l)	2,3	2.0	2.5	ns
HDL (mmol/l)	1.2	1.1	1.3	ns
CRP (mg/l)	3.4	3.9	3.1	ns
HbA1c (mmol/mol)	7.7	6.6	8.4	ns
Hb (g/dl)	139.7	139.5	139.8	ns

Table S2. Demographic data of the end-stage carotid atherosclerosis patients from the BiKE cohort used in plasma analysis.

Supplementary Table III

**General**	**Asymptomatic**	**Symptomatic**	**P-value**
N	40	87	
Age *(years, mean)*	**66.4**	**72.52**	**0.0002**
Sex *(male/female)*	**39/1**	**61/26**	**0.0003**
BMI *(mean)*	27,5	24,67	ns
Smoking *N yes (%)*	19 (47.5)	42 (48.3)	ns
**Symptoms**			
Minor stroke (MS)		32 (36.8)	
Transitory ischemic attack (TIA)		29 (33.3)	
Amaurosis fugax (AF)		26 (29.9)	
**Time (from symptom to surgery)**			
days <15		15 (17.2)	
15 to 30		24 (27.6)	
>30		48 (55.2)	
**Therapy**			
Lipid decreasers (ezetimib, HMG-CoA reductase inhibitors)	32 (80)	74 (85.1)	ns
Antidiabetics	10 (25)	20 (23)	ns
Antihypertensives (ACE inhibitors, beta-blockers, diuretics, ang II blockers)	34 (85)	76 (87.4)	ns
**Comorbidities**			
Previous myocardial infarction	6 (15)	20 (23)	ns
Inflammatory diseases (rheumatism, psoriasis, multiple sclerosis, inflammatory bowel disease)	1 (2.5)	3 (3.4)	ns
Hypertension	31 (77.5)	75 (86.2)	ns
Diabetes	10 (25)	22 (25.3)	ns
**Lab measurements** *(mean)*			
Serum creatinine (mg/dl)	89.54	97.64	ns
Serum cholesterol (mmol/l)	4,4	4.55	ns
LDL (mmol/l)	2.75	2.57	ns
HDL (mmol/l)	1	1.11	ns
CRP (mg/l)	9.2	15.86	ns
HbA1c (mmol/mol)	6.33	4,5	ns
Hb (g/dl)	**141.62**	**131.55**	**0.0077**

Table S3. Demographics of the BiKE patients used in plaque tissue microarray analysis

Supplementary Table IV

Cohort	Group (n)	Plasma OMD values (ng/ml) reported as mean ± SD
Chronic Kidney Disease	Grade 0 (n=24)	1.812 ± 0.904
	Grade 1 (n=25)	1.681 ± 0.718
	Grade 2 (n=24)	1.599 ± 0.507
	Grade 3 (n=22)	2.159 ± 0.966
Carotid atherosclerosis	Control (n=33)	3.466 ± 1.093
	Peripheral (n=88)	2.312 ± 2.128

Table S4. OMD protein values as quantified in the plasma of patients with CKD as well as carotid atherosclerosis and in a cohort of healthy subjects without reported CVD.

Supplementary Table V

Variable	Estimate	Standard error	95% CI	|t|	P-value
Intercept	1.054	4.379	-7.660 to 9.769	0.240	0.81
CALCVolProp	**0.057**	**0.025**	**0.006 to 0.109**	**2.242**	**0.02**
LRNCVolProp	0.056	0.034	-0.013 to 0.125	1.608	0.11
Plaque-burden volume ratio	1.925	10.07	-18.11 to 21.96	0.191	0.84
Wall to lumen volume ratio	-0.596	1.162	-2.909 to 1.717	0.513	0.60

Table S5. Multiple linear regression analysis of circulating OMD levels with CALCVolProp, LRNCVolProp, Plaque-burden volume proportion and Wall-to-lumen volume proportion (n=85 patients).
